# Obstructive Coronary Artery Disease Improved Prediction by the COME-CCT Pretest Probability Calculator With Cardiac CT

**DOI:** 10.1016/j.jacadv.2025.102014

**Published:** 2025-07-28

**Authors:** Viktoria Wieske, Mario Walther, Mahmoud Mohamed, Benjamin Weickert, Simon Andrzejewski, Benjamin Dubourg, Daniele Andreini, Gianluca Pontone, Hatem Alkadhi, Jörg Hausleiter, Mario J. Garcia, Sebastian Leschka, Willem B. Meijboom, Elke Zimmermann, Bernhard Gerber, U Joseph Schoepf, Abbas A. Shabestari, Bjarne L. Nørgaard, Matthijs FL. Meijs, Akira Sato, Kristian A. Øvrehus, Axel CP. Diederichsen, Shona M. Jenkins, Juhani Knuuti, Ashraf Hamdan, Bjørn A. Halvorsen, Vladimir Mendoza Rodriguez, Carlos Rochitte, Johannes Rixe, Yung-Liang Wan, Christoph Langer, Nuno Bettencourt, Eugenio Martuscelli, Said Ghostine, Ronny R. Buechel, Konstantin Nikolaou, Hans Mickley, Lin Yang, Zhaqoi Zhang, Marcus Y. Chen, David A. Halon, Matthias Rief, Kai Sun, Hiroyuki Niinuma, Roy P. Marcus, Simone Muraglia, Réda Jakamy, Benjamin JW. Chow, Philipp A. Kaufmann, Bernhard A. Herzog, Jean-Claude Tardif, Cesar Nomura, Klaus F. Kofoed, Jean-Pierre Laissy, Armin Arbab-Zadeh, Kakuya Kitagawa, Roger Laham, Masahiro Jinzaki, John Hoe, Frank J. Rybicki, Arthur Scholte, Narinder Paul, Swee Yaw Tan, Kunihiro Yoshioka, Robert Roehle, Georg M. Schuetz, Michael Laule, David E. Newby, Stephan Achenbach, Matthew Budoff, Robert Haase, Jonathan D. Dodd, Marc Dewey, Peter Schlattmann, COME-CCT Consortium

**Affiliations:** a Institute of Biometry and Clinical Epidemiology, Charité - Universitätsmedizin Berlin, Berlin, Germany; b Department of Fundamental Sciences, Jena University of Applied Sciences, Jena, Germany; c Cardiac Imaging Unit, Department of Radiology, Rouen University Hospital, Rouen, France; d Centro Cardiologico Monzino, IRCCS, Milan, Italy; e Institute of Diagnostic and Interventional Radiology University Hospital Zurich, Zurich, Switzerland; f Maximilians-University of Munich, Munich, Germany; g Department of Cardiology, Montefiore, University Hospital for the Albert Einstein College of Medicine, New York, New York, USA; h Department of Radiology, St Gallen, Switzerland; i Department of Cardiology, Erasmus University Medical Centre, Rotterdam, Netherlands; j Department of Cardiology, Clinique Universitaire St Luc, Institut de Recherche Clinique et Expérimentale, Brussels, Belgium; k Department of Radiology and Radiological Science, Medical University of South Carolina, Charleston, South Carolina, USA; l Modarres Hospital, Shahid Beheshti University of Medical Sciences, Tehran, Iran; m Department of Cardiology, Aarhus University Hospital, Aarhus, Denmark; n Department of Cardiology, University Medical Centre Utrecht, Utrecht, Netherlands; o Cardiovascular Division, Faculty of Medicine, University of Tsukuba, Tsukuba, Japan; p Department of Cardiology, Odense University Hospital, Odense, Denmark; q Glasgow Royal Infirmary and Stobhill Hospital, Glasgow, United Kingdom; r Turku University Hospital and University of Turku, Turku, Finland; s Cardiology Department, Rabin Medical Center, Petah Tikva, and Faculty of Medical and Health Sciences, Tel Aviv University, Tel Aviv, Israel; t Department of Cardiology, Ostfold Hospital Trust, Grålum, Norway; u Department of Cardiology, Woodlands Limited Hospital; Georgetown, Guyana; v Heart Institute, InCor, University of São Paulo Medical School, São Paulo, Brazil; w Department of Cardiology, Kerckhoff Heart Centre, Bad Nauheim, Germany; x Medical Imaging and Radiological Sciences, College of Medicine, Chang Gung University, Chang Gung Memorial Hospital at Linkou, Taoyaun, Taiwan; y Kardiologisch-Angiologische Praxis, Herzzentrum Bremen, Bremen, Germany; z Department of Cardiology, Centro Hospitalar de Vila Nova de Gaia, Vila Nova de Gaia, Portugal; aa Department of Internal Medicine, University of Rome Tor Vergata, Rome, Italy; ab Department of Cardiology, Centre Chirurgical Marie Lannelongue, Le Plessis Robinson, France; ac Department of Nuclear Medicine, University Hospital Zurich, Zurich, Switzerland; ad Department of Diagnostic and Interventional Radiology, University Hospital of Tübingen, Tübingen, Germany; ae Department of Radiology, Beijing Anzhen Hospital, Beijing, China; af National Heart and Blood Institute, National Institutes of Health, Bethesda, Maryland, USA; ag Cardiovascular Clinical Research Unit, Lady Davis Carmel Medical Center, Haifa, Israel; ah Department of Radiology, Baotou Central Hospital, Baotou, Inner Mongolia, China; ai St Luke’s International Hospital, Tokyo, Japan; aj Cantonal Hospital of Lucerne, Lucerne, Switzerland; ak Department of Cardiology, S Chiara Hospital, Trento, Italy; al Department of Cardiology, University Hospital Pitié-Salpêtrière, Paris, France; am University of Ottawa Heart Institute, Ottawa, Ontario, Canada; an Department of Nuclear Medicine, University Hospital Zurich, Zurich, Switzerland; ao HeartClinic Lucerne, Lucerne, Switzerland; ap Montreal Heart Institute, Université de Montréal, Montréal, Canada; aq Albert Einstein Hospital, São Paulo, Brazil; ar The Heart Centre, Rigshospitalet, University of Copenhagen, Copenhagen, Denmark; as Department of Radiology, Gonesse Hospital Center, Gonesse, France; at Division of Cardiology, Johns Hopkins Hospital, Johns Hopkins University, Baltimore, Maryland, USA; au Mie University Hospital, Tsu, Japan; av BIDMC/Harvard Medical School, Department of Cardiology, Beth Israel Deaconess Medical Center, Harvard University, Boston, Massachusetts, USA; aw Department of Radiology, Keio University Hospital, Tokyo, Japan; ax Department of Radiology, Mount Elizabeth Hospital, Singapore, Singapore; ay Department of Radiology, University of Cincinnati, Cincinatti, Ohio, USA; az Department of Cardiology, Leiden University Medical Centre, Leiden, Netherlands; ba Department of Medical Imaging, Western University, London, Ontario, Canada; bb National Heart Centre, Singapore, Singapore; bc Iwate Medical University, Morioka, Japan; bd Department of Cardiology, Charité - Universitätsmedizin Berlin, Berlin, Germany; be British Heart Foundation Centre of Research Excellence, University of Edinburgh, Edinburgh, United Kingdom; bf Department of Cardiology, Friedrich-Alexander University Erlangen-Nuremberg, Erlangen, Germany; bg Lundquist Institute, Harbor-UCLA Medical Center, Torrance, California, USA; bh Department of Radiology, St. Vincent’s University Hospital and School of Medicine, University College Dublin, Dublin, Ireland; bi Berlin Institute of Health, Berlin, Germany; bj DZHK (German Centre for Cardiovascular Research), Berlin, Germany; bk Institute of Medical Statistics, Computer Sciences and Data Science, University Hospital of Friedrich Schiller University Jena, Jena, Germany

**Keywords:** computed tomography angiography, coronary artery disease, disease probability, individual patient data meta-analysis, stable chest pain

## Abstract

**Background:**

Combining pretest probability (PTP) with computed tomography angiography (CTA) for diagnosing obstructive coronary artery disease (CAD) has not yet been determined.

**Objectives:**

The purpose of this study was to evaluate the accuracy of PTP calculation alone and with CTA for diagnosing CAD.

**Methods:**

A total of 65 prospective diagnostic accuracy studies of patients clinically referred to invasive coronary angiography with stable chest pain were included in this international collaborative individual patient data Collaborative Meta-Analysis of Cardiac CT (COME-CCT) meta-analysis. Mixed-effects logistic regression with a data set–specific random intercept for clustering was applied to 4 models: the traditional Diamond-Forrester models, a PTP model based on the COME-CCT data (termed COME-CCT-PTP calculator), a CTA alone model, and a combined COME-CCT-PTP with CTA model.

**Results:**

Individual patient data from 5,332 patients with clinically indicated invasive coronary angiography from 22 countries were included. The COME-CCT-PTP calculator was more accurate than the original Diamond-Forrester model (AUC: 0.68; 95% CI: 0.66-0.69 vs 0.63; 95% CI: 0.62-0.65). The COME-CCT-PTP with CTA model significantly improved accuracy compared with either model alone (AUC: 0.86; 95% CI: 0.85-0.87 vs 0.81; 95% CI: 0.80-0.82). The improved prediction was consistent in decision curve analysis with an increased net benefit for all chest pain subtypes and was almost equally seen in patients with typical or atypical angina (0.85; 95% CI: 0.84-0.86) and nonanginal or other chest discomfort (0.88; 95% CI: 0.86-0.89).

**Conclusions:**

Combining the COME-CCT-PTP calculator with CTA provides more accurate prediction than the PTP or CTA alone for the diagnosis of obstructive CAD, for all chest pain subtypes.

The prevalence of obstructive coronary artery disease (CAD) has decreased in men but increased in women, while disability-adjusted life years related to CAD are projected to increase to 2,275.9 worldwide by 2022.^1^ Stable chest pain is a common initial presentation of CAD^2^^,^^3^ and associated with the same increase in coronary mortality in women and men.^4^^,^^5^ Invasive coronary angiography (ICA) is the reference standard for the final diagnosis of obstructive CAD in patients with stable chest pain but decision-making about referral to ICA can be challenging.^6^ To facilitate appropriate referral to ICA in stable chest pain patients, American^7^ and European guidelines^8^ recommend pragmatic clinical prediction models for estimation of the probability of obstructive CAD. The American guideline recommends the updated contemporary models such as Juarez-Orozco et al^9^ while European guidelines recommend using age, sex, symptoms, and risk factors.^8^ The discriminative ability of these diagnostic prediction models has not been tested in large contemporary cohorts of patients clinically referred for ICA because of stable chest pain with suspected obstructive CAD.

We initiated the COME-CCT (Collaborative Meta-Analysis of Cardiac CT) Consortium to combine individual patient data from current diagnostic accuracy studies in which ICA served as the diagnostic reference standard and computed tomography angiography (CTA) was the index test in patients with stable chest pain suggestive of CAD. This allowed the calculation of a pretest probability (PTP) calculator, which we have termed the “COME-CCT-PTP calculator.”^10^ In this analysis, we investigated the discriminative ability of traditional PTP models such as the Diamond Forrester models compared with the COME-CCT-PTP calculator to identify patients with obstructive CAD. We also evaluated whether adding CTA results to the COME-CCT-PTP calculator resulted in a better prediction of obstructive CAD on ICA in patients with stable chest pain.

## Methods

The COME-CCT Consortium conducted an individual patient data meta-analysis of diagnostic accuracy data sets published between 2004 and 2014 that included patients with stable chest pain and suspected CAD in whom CTA was the index test and clinically indicated ICA served as the reference standard. The detailed protocol of the COME-CCT Consortium including the eligibility criteria, data collection, and harmonization and search strategy has been previously published^11^^,^^12^ and was registered in the PROSPERO Database for Systematic Reviews (CRD42012002780). For all retrieved studies, CTA results were blinded and thus did not influence the decision to perform ICA, avoiding verification bias. The ICA results were blinded to authors assessing clinical predictors and to CTA readers. The results from the international individual patient data meta-analysis were used to calculate a PTP calculator, termed the “COME-CCT-PTP calculator.” Reporting of our study follows the TRIPOD (Transparent Reporting of a multivariable prediction model for Individual Prognosis or Diagnosis) statement^13^ (see the TRIPOD checklist in the Supplemental Appendix) and the suggestions of Debray et al regarding diagnostic prediction models using individual participant data meta-analysis.^14^ Quality assessment of published articles was performed using the QUADAS (quality assessment tool for diagnostic accuracy studies) and the STARD (Standards for Reporting of Diagnostic Accuracy) checklist, and results were recently published on the study-level.^15^^,^^16^ We used the methodology described by Wasson and Sox in developing the COME-CCT diagnostic prediction models.^17^ This meta-analysis did not require Institutional Board Review as it involved the secondary analysis of publicly available, deidentified data.

### CAD prediction models

Three CAD prediction models were built by using a mixed effects logistic regression model based on the COME-CCT data with a data set–specific random intercept to account for clustering.^18^ For the COME-CCT-PTP calculator, typical angina was considered when the following 3 criteria were fulfilled: retrosternal chest discomfort, precipitation by exertion, and prompt relief (within 30 s–10 min) by rest or nitroglycerin.^19^ Patients who met 2, one, or none of these 3 criteria were classified as having atypical angina, nonanginal chest discomfort, and other chest discomfort, respectively. The category “other chest discomfort” was used as recommended^20^ since these patients were not asymptomatic but had chest pain not fulfilling any of the 3 criteria. These criteria are also used in the Diamond-Forrester prediction models recommended in previous European guidelines.^21^ To assess whether CTA alone can be used as an accurate predictor of CAD, we performed a second logistic regression model incorporating CTA alone.^22^ Finally, to assess whether a combination of the COME-CCT-PTP calculator combined with CTA results was a better predictor than either model alone, we performed a third logistic regression model incorporating CTA as an additional covariate.

### Statistical analysis

Individual patient data were analyzed using a mixed effects logistic regression model with a data set–specific random intercept to take clustering within studies into account.^18^ No imputation methods were used and only patients with complete information required for pragmatic prediction models were included. The binary outcome “obstructive CAD” was defined in all retrieved studies as at least one ≥50% diameter stenosis by ICA. The analysis was performed including nondiagnostic CTA results in the model using 2-by-3 tables.^23^ For nondiagnostic CTA results, we assumed a worst-case scenario, that is, for an ICA negative result, we defined a corresponding nondiagnostic CTA result to be CTA positive, and an ICA positive result with a nondiagnostic CTA was defined as CTA negative. All covariates (age, sex, chest pain type, and CTA) in the regression models were included as main effects. No interaction terms were used. The incremental gain from using CTA results in estimating CAD probability combined with the COME-CCT-PTP calculator was evaluated by reduction of the Bayesian information criterion. Disease probabilities were predicted by averaging over the random-effects distribution.^24^ A sensitivity analysis of the predictions was performed using an average intercept applied over all studies.^25^ Potential publication bias was assessed as described previously.^12^ Overall performance of the models for predicting CAD probability was evaluated by the scaled Brier score, which measures the accuracy of probabilistic prediction.^26^ A perfect model results in a Brier score of zero or a scaled Brier score of one. Discriminative ability of the models, that is, the ability of discriminating patients with obstructive CAD from those without, was quantified using the area under the receiver-operating characteristic curve (AUC) with 95% CIs.^27^ Calibration as the extent of disagreement and bias with respect to observed and predicted outcomes was investigated graphically based on calibration plots. We also evaluated all 3 prediction models in terms of their benefit in clinical practice using decision curve analysis.^28^

Internal model validation for the models with and without CTA was based on 250 bootstrap samples of the original data,^29^ using AUC, scaled Brier score, accuracy, and the discrimination slope,^30^ which was calculated as the absolute difference in average predictions for those with and without CAD. Furthermore, the COME-CCT-PTP calculator was compared with the original Diamond-Forrester^31^ and updated Diamond-Forrester prediction models^32^ using the same 3 clinical variables and the published coefficients of the respective models. Comparison with the original Diamond-Forrester model included 4,099 patients aged 30 to <70 years while the updated Diamond-Forrester model included all age groups but included nonanginal or other chest discomfort. We applied internal-external cross-validation as described,^25^ where the ratio of expected and observed events and also the calibration slope were considered. The average calibration slope was calculated using a random-effects meta-analytical method with inverse variance as weights.^33^ All computations were performed with the statistical software R^34^ using lme4 package^35^ to apply generalized linear mixed models and PredictABEL package^36^ for evaluating model calibration and discrimination and meta^37^ for investigating averaged calibration slopes. Decision curve analysis was run using the dca-function of R.

## Results

**Figure 1:**
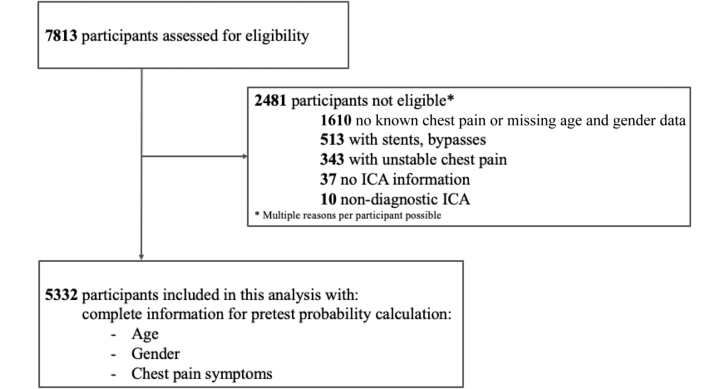
**Eligibility Assessment and Inclusion** Patients with stable chest pain, complete information on age, sex, and chest pain symptom, without known CAD and information on ICA and CTA were available for inclusion.^12^ CAD = coronary artery disease; CTA = computed tomography angiography; ICA = invasive coronary angiography.

A total of 5,332 stable chest pain patients with clinically indicated ICA from 22 countries were included, see Figure 1. Number of patients with (2,573) and without obstructive CAD (2,759) was almost equal with a CAD prevalence of 48.3%. Clinical and technical characteristics of the studies are shown in Table 1, and general study characteristics can be found in Supplemental Table 1. Participant characteristics for each study, further study characteristics, and technical characteristics of imaging testing can be found in the COME-CCT main analysis publication.^12^ The median age was 61 years, about 2-thirds were male, and the majority of patients had either typical or atypical angina.

**Table 1:** Participant Characteristics

	Obstructive CAD (n = 2,573)	No CAD (n = 2,759)	Total (n = 5,332)
Age (in y), median (IQR)	63 (56-70)	60 (53-67)	61 (54-69)
Male	1947 (75.7%)	1,526 (55.3%)	3,473 (65.1%)
Chest pain symptoms			
Typical angina	1,199 (46.6%)	768 (27.8%)	1967 (36.9%)
Atypical angina	641 (24.9%)	951 (34.5%)	1,592 (29.9%)
Nonanginal chest discomfort	324 (12.6%)	472 (17.1%)	796 (14.9%)
Other chest discomfort	409 (15.9%)	568 (20.6%)	977 (18.3%)
Risk factor distribution, n/N (%)			
Arterial hypertension	1,442/2,374 (60.7%)	1,207/2,512 (48.1%)	2,649/4,886 (54.2%)
Diabetes mellitus	559/2,379 (23.5%)	347/2,517 (13.8%)	906/4,896 (18.5%)
Hyperlipidemia	1,322/2,242 (59.0%)	1,079/2,326 (46.4%)	2,401/4,568 (52.6%)
Smoker	697/2,378 (29.3%)	592/2,519 (23.5%)	1,289/4,897 (26.3%)
Former smoker	455/1793 (25.4%)	442/1949 (22.7%)	897/3,742 (24.0%)
Positive family history	911/2,212 (41.2%)	889/2,346 (37.9%)	1800/4,558 (39.5%)
CT detector rows, n/N (%)			
16	583/2,504 (23.3%)	674/2,720 (24.8%)	1,257/5,224 (24.1%)
23	368/2,504 (14.7%)	381/2,720 (14.0%)	749/5,224 (14.3%)
40	77/2,504 (3.1%)	145/2,720 (5.3%)	222/5,224 (4.3%)
64	1,219/2,504 (48.7%)	1,219/2,720 (44.8%)	2,438/5,224 (46.7%)
128	100/2,504 (4.0%)	127/2,720 (4.7%)	227/5,224 (4.4%)
320	157/2,504 (6.3%)	174/2,720 (6.4%)	331/5,224 (6.3%)
CTA showing obstructive CAD^b^	2,251 (87.5%)	728 (26.4%)	2,979 (55.9%)
Information on effective dose	1935 (75.2%)	2073 (75.1%)	4,008 (75.2%)
Effective dose (in mSv), mean (SD)	13.27 (6.9) (n = 1935)	13.5 (7.8) (n = 2073)	13.39 (7.4) (n = 4,008)

Figures are numbers (percentage) of patients unless stated otherwise^a^.

CAD = coronary artery disease; CTA = computed tomography angiography; CT = computed tomography.

a Additional study characteristics can be found in Supplemental Table 1.

b Obstructive CAD was defined as $50% coronary diameter stenosis by CTA and ICA.

### COME-CCT-PTP calculator alone

**Figure 2:**
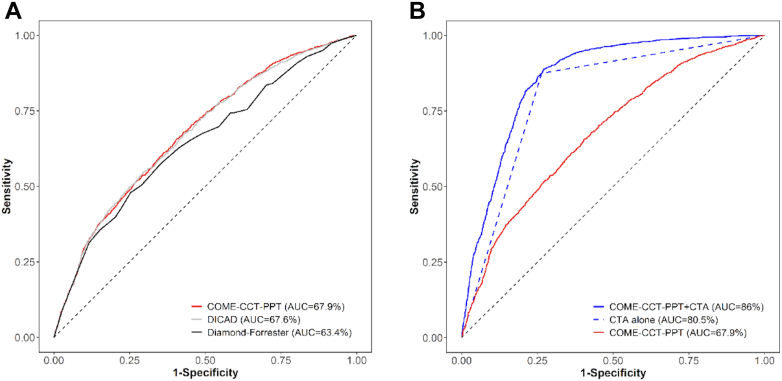
**Discriminative Ability of Prediction Models** Panel (A) shows the receiver-operating characteristic (ROC) curves of the clinical probability prediction (PTP) models. The COME-CCT-PTP calculator (red) and updated Diamond-Forrester model (gray) resulted in significantly improved discrimination compared to the original Diamond-Forrester model (black). Panel (B) shows the significantly improved discriminative ability using the combination of the COME-CCT-PTP calculator with CTA (solid blue) compared to CTA alone (dashed blue). COME-CCT = Collaborative Meta-Analysis of Cardiac CT; DICAD = updated Diamond-Forrester model; other abbreviation as in Figure 1.

The COME-CCT-PTP calculator improved discrimination of patients with suspected CAD with an AUC of 0.68 (95% CI: 0.66-0.69) compared with the original Diamond-Forrester prediction model (AUC = 0.63 [95% CI: 0.62-0.65]) (Figure 2A) but not when compared with the updated Diamond-Forrester prediction model (AUC = 0.68 [95% CI: 0.66-0.69]). Table 2 shows the COME-CCT-PTP calculator results according to age, sex, and chest pain type combined with CTA results. Data set–specific results are shown in Supplemental Table 3. Internal validation of the COME-CCT-PTP calculator showed minimal optimism bias, indicating valid estimates of performance (Supplemental Table 4). Internal-external cross-validation showed a median AUC of 0.72 (IQR: 0.62-0.80) (Supplemental Table 5). The meta-analytic average calibration slope was 1.13 (95% CI: 0.94-1.32) and a median ratio of expected/observed results of 1.05 (IQR: 0.81-1.31) (Supplemental Table 5). Supplemental Figure 1 presents a graphic analysis of calibration of the pragmatic clinical prediction model. For the average intercept model in the sensitivity analysis, we obtained an average calibration slope of 1.00 (95% CI: 0.83-1.16) (Supplemental Table 6).

**Table 2:** COME-CCT-PTP Calculator Results According to Age, Sex, Classification of Chest Pain Type, and Stratified by CTA Results Showing Obstructive (+) and Nonobstructive CAD (−) Based on the Combined Prediction Model and CTA

Age, y	Typical Angina		Atypical Angina		Nonanginal Chest Discomfort		Other Chest Discomfort	
	Women	Men	Women	Men	Women	Men	Women	Men
20 CT+/−	21 50/6	39 69/13	9 26/2	19 44/5	7 25/2	17 43/4	7 22/2	15 39/4
25 CT+/−	24 54/7	43 72/15	10 29/2	22 47/5	9 28/2	19 46/5	8 25/2	17 43/4
30 CT+/−	27 57/8	47 75/16	12 31/3	25 51/6	10 31/3	22 50/6	9 28/2	20 46/5
35 CT+/−	30 60/9	51 77/18	14 34/3	28 54/7	12 34/3	25 53/7	11 31/3	23 49/6
40 CT+/−	34 64/10	55 80/21	16 38/4	31 57/8	14 37/3	28 56/8	12 34/3	26 53/7
45 CT+/−	37 67/12	59 82/23	18 41/4	35 61/9	16 40/4	31 60/9	14 37/3	29 56/8
50 CT+/−	41 70/13	62 84/26	21 44/5	38 64/10	18 43/5	34 63/10	16 40/4	32 60/9
55 CT+/−	45 73/15	66 86/28	23 48/5	42 67/12	20 47/5	38 66/11	19 43/5	36 63/10
60 CT+/−	49 75/17	69 87/31	26 51/6	46 70/13	23 50/6	42 69/13	21 47/5	39 66/11
65 CT+/−	53 78/19	73 89/34	30 55/7	50 73/15	26 54/7	46 72/15	24 50/6	43 69/13
70 CT+/−	57 80/21	76 90/38	33 58/8	54 76/17	29 57/8	50 75/16	27 54/7	47 72/15
75 CT+/−	60 82/24	79 91/41	37 61/9	58 78/19	33 60/9	54 77/19	31 57/8	51 75/16
80 CT+/−	64 84/26	81 93/44	40 65/11	61 80/21	36 64/10	57 80/21	34 60/9	55 77/18
85 CT+/−	68 86/29	84 93/48	44 68/12	65 83/24	40 67/12	61 82/23	38 64/10	59 80/21
90 CT+/−	71 88/32	86 94/51	48 71/14	69 84/26	44 70/13	65 84/26	41 67/12	63 82/23
95 CT+/−	74 89/35	88 95/54	52 73/15	72 86/29	48 73/15	68 86/28	45 70/13	66 84/26
100 CT+/−	77 91/38	89 96/58	56 76/17	75 88/32	52 75/17	72 87/31	49 73/15	70 86/28
105 CT+/−	80 92/41	91 96/61	60 79/20	78 89/35	56 78/19	75 89/34	53 75/17	73 87/31

COME-CCT = Collaborative Meta-Analysis of Cardiac CT; PTP = pretest probability; other abbreviations as in Table 1.

Numbers are percentages unless otherwise stated. Typical angina defined as retrosternal chest discomfort, precipitation by exertion, and prompt relief (within 30 s-10 min) by rest or nitroglycerin.^19^ Patients who met 2, 1, or 0 of these 3 criteria were classified as having atypical angina, nonanginal chest discomfort, and other chest discomfort, respectively.

### CTA alone

**Figure 3:**
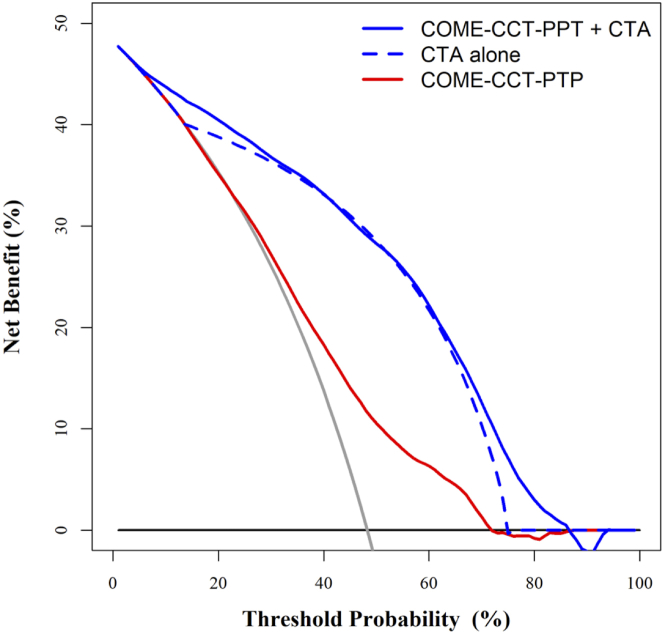
**Decision Curves of the COME-CCT-PTP Calculator Alone, CTA Alone, and Combined COME-CCT-PTP Calculator and CTA** The figure shows the net benefit of the COME-CCT-PTP calculator alone (red), combined with CTA (blue) and CTA alone (dashed blue) as a function of threshold probability. Patients are classified as test-positive (diagnosed with obstructive CAD) or test-negative (no CAD) based on their CAD probability. The net benefit based on the CTA alone model performed better than the COME-CCT-PTP calculator, and further improved by the combination. The black line represents the net benefit of considering all patients as having no CAD; the gray line represents the net benefit of considering all patients to have obstructive CAD. The intersection of the black with the gray line indicates the prevalence of obstructive CAD (48.3%) in our patient sample. PTP = pretest probability; other abbreviations as in Figure 1, Figure 2.

Using CTA alone resulted in an AUC to predict CAD of 0.81 (95% CI: 0.80-0.82) (Figure 2B). Data set–specific results are shown in Supplemental Table 7. The CTA alone model showed improved stratification of patients with and without obstructive CAD compared to the COME-CCT-PTP calculator (Supplemental Figures 2 and 3). Internal validation of prediction of the CTA alone model showed minimal optimism bias indicating valid estimates of the performance (Supplemental Table 4). Decision curve analysis regarding net benefit of the CTA alone model showed improved discrimination compared with the COME-CCT-PTP calculator (Figure 3). Internal-external cross-validation showed a median AUC of 0.86 (IQR: 0.79-0.93) (Supplemental Table 8). We found a meta-analytic average calibration slope of 1.13 (95% CI: 0.94-1.32). For the average intercept model in the sensitivity analysis, we obtained an average calibration slope of 1.00 (95% CI: 0.83-1.16) (Supplemental Table 6).

### COME-CCT-PTP calculator with CTA

Combining CTA with the COME-CCT-PTP calculator improved CAD probability prediction compared with either alone, and resulted in a good discriminative ability with an AUC of 0.86 (95% CI: 0.85-0.87) (Figure 2B) (Table 3). The heterogeneity measured by the variance component estimate was reduced from 0.667 to 0.377 when CTA was combined with the COME-CCT-PTP calculator (Table 3), indicating 43.5% of the variability between sites was explained by CTA. Data set–specific results are shown in Supplemental Table 10. The addition of CTA to the COME-CCT-PTP calculator improved stratification of patients with and without obstructive CAD (Supplemental Figures 2 and 3). Data set–specific results of the AUC (Supplemental Table 10) showed a substantial decrease in heterogeneity between studies when CTA was combined with the COME-CCT-PTP calculator model (Supplemental Figure 4). Internal validation of prediction including CTA showed minimal optimism bias indicating valid estimates of performance (Supplemental Table 4). Decision curve analysis regarding net benefit of including CTA in prediction showed markedly improved discrimination compared with the COME-CCT-PTP calculator alone (Figure 3). Internal-external cross-validation showed a median AUC of 0.91 (IQR: 0.84-0.95) (Supplemental Table 11). There was an average calibration slope of 1.28 (95% CI: 1.09-1.47) and a median ratio of expected/observed results of 1.01 (IQR: 0.90-1.19) (Supplemental Table 11). For the average intercept model in the sensitivity analysis, there was an average calibration slope of 1.19 (95% CI: 1.02-1.37) (Supplemental Table 12). Supplemental Figure 5 presents a graphic analysis of calibration of the pragmatic clinical prediction model with CTA.

**Table 3:** Estimates of the Logistic Regression Models With Random Effects (N = 5,332)

	Estimate (SE)	*P* Value	OR (95% CI)
COME-CCT-PTP calculator			
Age (centered)	0.036 (0.003)	<0.001	1.04 (1.03-1.04)
Sex (male vs female)	0.986 (0.068)	<0.001	2.68 (2.35-3.06)
Symptoms^a^			
Typical angina	1.427 (0.118)	<0.001	4.17 (3.30-5.25)
Atypical angina	0.307 (0.117)	0.009	1.36 (1.08-1.71)
Nonanginal chest discomfort	0.119 (0.132)	0.365	1.13 (0.87-1.46)
Other chest discomfort	Reference group		1.00
Model constant	−1.434 (0.151)	―	―
Random intercept (τ^2^)^b^	0.667 (0.816)		
BIC	6,398.43		
logLik	−3,169.18		
CTA			
CTA (obstructive vs nonobstructive CAD)	2.973 (0.078)	<0.001	19.55 (16.78-22.78)
Model constant	−1.893 (0.103)	―	―
Random intercept (τ^2^)^b^	0.348 (0.590)		
BIC	5,061.43		
logLik	−2,517.84		
Combined COME-CCT-PTP calculator with CTA			
Age (centered)	0.030 (0.004)	<0.001	1.03 (1.02-1.04)
Sex (male vs female)	0.868 (0.081)	<0.001	2.38 (2.03-2.79)
Symptoms^a^			
Typical angina	1.346 (0.137)	<0.001	3.84 (2.94-5.03)
Atypical angina	0.195 (0.134)	0.146	1.22 (0.93-1.58)
Nonanginal chest discomfort	0.156 (0.154)	0.310	1.17 (0.86-1.58)
Other chest discomfort	Reference group		1.00
CT result (obstructive vs not obstructive)	2.924 (0.082)	<0.001	18.62 (15.86-21.86)
Model constant	−3.030 (0.159)	―	―
Random intercept (τ^2^)^b^	0.377 (0.614)		
BIC	4,740.58		
logLik	−2,335.96		

BIC = Bayesian information criterion; other abbreviations as in Table 1, Table 2.

a Typical angina defined as retrosternal chest discomfort, precipitation by exertion, and prompt relief (within 30 s-10 min) by rest or nitroglycerin. Patients in whom 2, one, or none of these 3 criteria were found were classified as having atypical angina, nonanginal chest discomfort, and other chest discomfort, respectively.

b Variance component estimate (τ2) for random intercept. Please see Supplemental Table 2 for the published coefficients of the updated Diamond-Forrester model. For the original Diamond-Forrester model we used the published look-up tables.^31^

### Subgroup analyses

The discriminative ability of the COME-CCT-PTP calculator decreased with less typical chest pain (typical or atypical angina (0.70; 95% CI: 0.68-0.71) vs nonanginal or other chest discomfort (0.63; 95% CI: 0.60-0.65) (Central Illustration). The improved prediction of CAD by combining CTA with the COME-CCT-PTP calculator prediction model was consistent in decision curve analysis with an increased net benefit for all chest pain types and was almost equally seen in patients with typical or atypical angina. Similar improvement was seen in terms of AUC in patients with typical or atypical angina (0.85; 95% CI: 0.84-0.86) and nonanginal or other chest discomfort (0.88; 95% CI: 0.86-0.89) (Central Illustration). Results stratified by symptoms were similar in the sensitivity analysis (Supplemental Tables 13 to 18). For the prediction model including CTA alone, the discriminative ability in the subgroups was decreased compared to the combined model: for patients with typical or atypical angina (0.79; 95% CI: 0.77-0.80) vs nonanginal or other chest discomfort (0.85; 95% CI: 0.83-0.86).

**Central Illustration:**
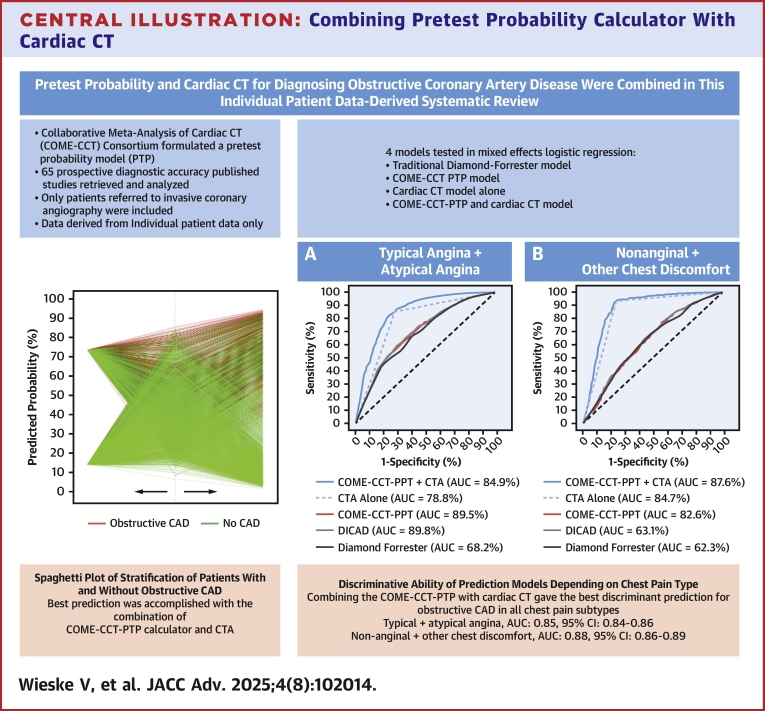
**Combining Pretest Probability Calculator With Cardiac CT** Abbreviations as in Figure 1, Figure 2.

Similar analysis for other subgroups including sex, age, and CAD prevalence showed similar results. For sex, the COME-CCT-PTP calculator with CTA improved discrimination (AUC = 0.85; 0.84-0.86) compared with the CTA alone model (AUC = 0.80; 0.79-0.81) and COME-CCT-PTP calculator alone (AUC = 0.65; 0.63-0.66) for both sexes (Supplemental Figure 6). For age, the COME-CCT-PTP calculator with CTA improved discrimination (AUC = 0.86; 0.84-0.87) compared with the CTA alone model (AUC = 0.80; 0.79-0.81) and the COME-CCT-PTP calculator alone (AUC = 0.67; 0.66-0.68) (Supplemental Figure 7). The COME-CCT-PTP calculator with CTA performed slightly better in patients < 50 years of age. For CAD prevalence, the COME-CCT-PTP calculator alone, CTA alone, and combined all performed better in patients with low CAD prevalence compared to the higher CAD prevalence (Supplemental Figure 8).

## Discussion

In comparison to ICA as the reference standard for CAD, our analysis showed pragmatic clinical prediction models to have limited discriminative ability in patients with stable chest pain suggestive of CAD. Combining clinical probability calculators with CTA improved discriminative ability compared to either alone, showing added value especially for patients with typical and atypical angina pectoris with improved patient stratification. The principal findings are immediately relevant for the diagnostic management of patients presenting with stable chest pain because clinically predicting whether or not a patient has obstructive CAD is pivotal but challenging.^6^

The Cochrane individual patient data meta-analysis methods group showed that individual patient data are being underutilized to facilitate guideline drafting and application.^38^ The current analysis has great clinical potential to improve existing guidelines in patients with stable chest pain suggestive of obstructive CAD^7^^,^^22^ by ensuring that routine patient care is based on the most reliable evidence available.^38^ Whether pragmatic clinical prediction of PTP, as recommended by the U.S. American and European guidelines, should always precede CTA or whether patients with stable typical or atypical angina should undergo direct CTA, as recommended in the updated National Institute for Health and Care Excellence clinical guideline 95, is an important question. We believe our data show that the combination of PTP and CTA provides the greatest clinical value.

While updated guidelines use PTP models provide much lower predicted rates of obstructive CAD, we were unable to use the updated European Society of Cardiology (ESC) 2019 PTP calculator^22^ as it includes dyspnea as a variable in the score and most retrieved papers did not provide a record of these data on an individual patient data level. We wanted a fair comparison to the Genders PTP model^32^^,^^39^ in similar cohorts with similar disease prevalences so that is why we included the Diamond-Forrester PTP models. Furthermore, the COME CCT individual patient data were collected from trials between 2004 and 2014 with an obstructive CAD prevalence of 48.3%, the closest CAD prevalence to the Diamond-Forrester and the updated Diamond-Forrester prediction models used in the 2013 ESC guidelines.^21^ The 2019 ESC PTP calculations were updated due to the decrease in obstructive CAD prevalence. The 2024 ESC PTP included coronary artery calcium score which does not provide an assessment of noncalcified plaque (a major predictor of major adverse cardiac events in contemporary cardiac CT trials such as SCOT-HEART [Scottish COmputed Tomography of the HEART] and DISCHARGE [Diagnostic Imaging Strategies for Patients with Stable Chest Pain and Intermediate Risk of Coronary Artery Disease]).^10^^,^^40^ The 2024 guideline compared their prevalences to external CTA cohorts rather than to the current clinical gold standard of ICA for obstructive CAD as derived from the current paper. The 2024 guidelines did not include CT in their Risk Factor-weighted Clinical Likelihood model. Our analysis shows the added value of the PTP calculations even in the presence of CTA results, which advocates for the use of PTP. In clinical practice, despite patients having a low PTP, many patients may still and do undergo ICA with negative results.^41^^,^^42^ Our future analysis will apply the COME-CCT-PTP with CTA in the DISCHARGE trial cohort to provide external validation in a pragmatic cohort for such a proposed model.

The COME-CCT-PTP calculator model with CTA provides individual pretest and posttest probability assessment for clinical decision-making, for example, when posttest probability after negative CTA is low (<15% according to the European guideline), the patient should be evaluated for other underlying causes of chest pain. In patients with intermediate PTP of CAD, a positive CTA is likely to increase posttest probability, and ICA is recommended in high-risk anatomy CAD. The prediction model uses information routinely available during clinical evaluation (age, sex, and chest pain characteristics) and thus may result in more frequent use^43^ than more time-consuming approaches such as the Duke clinical score, which requires 9 patient characteristics for probability estimation.^44^ Our COME-CCT-PTP calculator model combined with CTA is a pragmatic decision aid as recommended by American^7^ and European guidelines,^22^ aimed at improving physician-patient interactions by providing immediate evidence-based feedback on the probability of CAD according to the Salzburg statement.^45^ Most importantly, the probabilities provided by the prediction model may facilitate better integration of disease probabilities and risks of complications with patients’ preferences for subsequent testing^46^ into shared decision-making processes.^47^ This has potential to increase cost-effectiveness and patient safety by reducing unnecessary testing.^48^

The discriminative ability of pragmatic clinical prediction models decreases in patients with nonanginal or other chest discomfort when compared with patients presenting with typical or atypical angina pectoris. This is clinically important as more accurate prediction is needed in patients with less typical presentation because ambiguity about appropriate diagnostic testing and management is greatest in these patients with intermediate disease probability. About a third of the cohort in our analysis had nonanginal or other chest discomfort and in this group the added value of CTA for improving the prediction was most evident. Including CTA with the PTP model, resulting in an AUC of 0.86, clearly improved differentiation of patients with stable chest pain and suspected CAD compared with PTP models alone. Winther et al recently showed that more patients were reclassified with low PTPs (<15%) using a large Danish chronic coronary syndrome cohort, of which 8,028 patients (19%) underwent both CTA and ICA correlation.^49^ However, PTP models were not combined with CTA in that analysis, and instead CTA and ICA were used as a combined endpoint. Cheng et al studied a population of 14,048 patients clinically referred for CTA, but not ICA, and showed that CAD prevalence was overestimated by three-fold (51% vs 18%) using the original Diamond-Forrester prediction model for estimating probabilities.^50^ The better agreement in our study can be explained by a higher-risk population with 48.3% prevalence of CAD, and all patients had already been clinically referred for ICA. Hence, among lower-risk patients, such as the subgroup of nonanginal or other chest discomfort patients in our study and a recent single-center analysis of 2,274 patients referred for CT,^51^ clinical prediction alone becomes less accurate because it is being applied to populations from which the predictors were not derived. Importantly, the good discriminative ability of the COME-CCT-PTP calculator with CTA was similar in patients with typical or atypical angina and in patients with nonanginal or other chest discomfort. Including coronary calcium score by CT improves clinical prediction but was not included as discriminative ability and noncalcified plaque burden assessment is less compared with CTA.^39^ We also decided against the inclusion of functional testing into the prediction model because of limited sensitivity when adjusted for referral bias.^52^ Indeed, Patel et al have recently shown that the diagnostic yield of ICA is highest if it is performed following CTA (around 70%) and is relevantly lower (around 45%) when following by any functional test.^53^ In a smaller study of 527 patients with acute-onset chest pain, functional tests did not improve discriminative ability.^54^ Since both CTA and functional tests showed advantages in the PROMISE (PROspective Multicenter Imaging Study for Evaluation of Chest Pain)^55^ and SCOT-HEART trial,^56^ it would be interesting to compare their value for refining disease risk estimations.

### Study limitations

Patients included in the COME-CCT Consortium had an indication for ICA, which was performed in all patients to avoid verification bias. The cohort thus represents a certain spectrum of patients with suspected CAD with a prevalence of obstructive CAD of 48.3% and only 33.3% of patients with nonanginal or other chest discomfort. Results may not be representative of lower-risk patients being considered for CTA.^57^ Moreover, the reference standard ICA has considerable interobserver variability, although quantitative analysis of ICA was used in more than 70% of retrieved studies.^58^ Invasive fractional flow reserve is an alternative reference standard for assessing the functional significance of anatomic lesions but was not consistently determined in retrieved studies. CT perfusion^59^ and CT fractional flow reserve^60^ are recent tools for quantitative assessment of myocardial ischemia,^61^ but not yet widely available in clinical practice. As commonly observed in individual patient data meta-analyses,^62^ only 49% of individual patient data could be obtained from all original studies. Moreover, meta-analyses with inclusion of more than 50 original studies, such as ours, are rare and more difficult to conduct.^62^ Nondiagnostic CTA results were included in the model using 2-by-3 tables^23^ as a sensitivity analysis and showed slightly reduced discriminative ability especially in patients with typical or atypical angina. The clinical prediction including CTA showed clinical net benefit in the decision curve analysis and good predictive performance with good discriminative ability and acceptable calibration. The internal-external cross-validation showed acceptable results. Future work should focus in large cohort trials on validating a combined PTP and CTA approach to optimize patient management and treatment.

## Conclusions

Our collaborative analysis of individual patient data shows the potential for combining clinical PTP assessments with CTA, which may result in improved clinical decision-making in patients with suspected CAD.

Perspectives **COMPETENCY IN MEDICAL KNOWLEDGE:** Pragmatic clinical prediction models have a poor discriminative ability to predict obstructive CAD, particularly in patients with nonanginal or other chest discomfort. Incorporating cardiac CT results into such models may significantly improve the differentiation of patients with stable chest pain and obstructive disease.

**TRANSLATIONAL OUTLOOK:** Adding cardiac CTA to clinical prediction models may improve their discriminative ability in detecting patients with obstructive CAD and should be evaluated in future validation trial cohorts.

## Funding support and author disclosures

The COME-CCT Consortium is funded by a joint program of the 10.13039/501100001659German Research Foundation and the German Federal Ministry of Education and Research (01KG1110) and the Digital Health Accelerator of the Berlin Institute of Health to Dr Dewey. All researchers are independent of the funding bodies. The funding bodies had no role in the study design; in the collection, analysis, and interpretation of data; in the writing of the report; and in the decision to submit the manuscript for publication. Dr Wieske has received grant support from the FP7 Program of the European Commission for the randomized multicenter DISCHARGE trial (603266-2, HEALTH-2012.2.4.-2). Dr Pontone has received other grants from 10.13039/100004313General Electric, grants from 10.13039/100004313General Electric, other from 10.13039/100004374Medtronic, other from Bracco, outside the submitted work. Dr Hoe is on the Speakers Bureau for Abbott Vascular and Edwards Lifesciences. Dr Gerber reports that the Cliniques St Luc UCL holds a master research agreement with Philips Medical Systems. Dr Schoepf has received institutional grants, personal fees, and nonfinancial support from Astellas, 10.13039/100004326Bayer, 10.13039/100004313General Electric, 10.13039/100020333Guerbet, 10.13039/100020588HeartFlow, and 10.13039/100004340Siemens. Dr Nørgaard has received grants from 10.13039/100004340Siemens and 10.13039/100020588HeartFlow. AS has received personal fees from General Electric and Toshiba. Dr Knuuti has received grants from CardiRad and personal fees from GE Healthcare. Dr Buechel reports that the University Hospital Zurich holds a research contract with GE Healthcare. Dr Nikolaou reports collaborations with and project funding from Siemens Healthineers, Bayer Healthcare, GE Healthcare, and Speakers Bureau: Siemens Healthineers, Bayer Healthcare. Dr Chen has received an institutional research agreement with Canon Medical, formerly Toshiba Medical (no financial support/funding). Dr Halon has received other grant from Philips Healthcare, Cleveland, Ohio, during the conduct of the primary study. Dr Chow holds the Saul and Edna Goldfarb Chair in Cardiac Imaging Research; has received research support from GE Healthcare and educational support from TeraRecon Inc during the conduct of the study. Dr Kaufmann reports that the University Hospital Zurich holds a research agreement with GE Healthcare. Dr Arbab-Zadeh has received grant support from Canon Medical Systems. Dr Paul is on the Speakers Bureau for Toshiba Medical Systems; and has received grants from Toshiba Medical Systems, outside the submitted work. Dr Schuetz has received grants support for his salary from German Federal Ministry of Education and Research (BMBF) during the conduct of the study. Dr Dewey has received grant support from the FP7 Program of the European Commission for the randomized multicenter DISCHARGE trial (603266-2, HEALTH-2012.2.4.-2); also has received grant support from 10.13039/501100001659German Research Foundation (DFG) in the Heisenberg Program (DE 1361/14-1), graduate program on quantitative biomedical imaging (BIOQIC, GRK 2260/1), for fractal analysis of myocardial perfusion (DE 1361/18-1), the Priority Programme Radiomics for the investigation of coronary plaque and coronary flow (DE 1361/19-1 [428222922] and 20-1 [428223139] in SPP 2177/1); and also received funding from the Berlin University Alliance (GC_SC_PC 27) and from the Digital Health Accelerator of the Berlin Institute of Health. Dr Dewey has received lecture fees from Canon, Guerbet. Prof Dodd has received grant support from the Irish Lung Foundation, the St. Vincent’s Hospital Group Foundation, University College Dublin, and the FP7 Program of the European Commission for the randomized multicenter DISCHARGE trial (603266-2, HEALTH-2012.2.4.-2); is an associate editor of *Radiology*, *Respirology*, and the *Quarterly Journal of Medicine*; is an Editorial Board member of Radiology Cardiothoracic Imaging; and is an author in the Stat-Dx book Series Diagnostic Imaging – Cardiovascular and the textbook CT and MRI in Cardiology, Elsevier and the opinions expressed in this article are the author’s own and do not represent the view of ESR. Per the guiding principles of ESR, the work as Research Chair is on a voluntary basis and only remuneration of travel expenses occurs. Dr Dewey is also the editor of *Cardiac CT*, published by Springer Nature, and offers hands-on courses on CT imaging (www.ct-kurs.de). Institutional master research agreements exist with Siemens, General Electric, Philips, and Canon. The terms of these arrangements are managed by the legal department of Charité–Universitätsmedizin Berlin. Dr Dewey holds a joint patent with Florian Michallek on dynamic perfusion analysis using fractal analysis (PCT/EP2016/071551). All other authors have reported that they have no relationships relevant to the contents of this paper to disclose.
