# Supplementary material for: Obstructive Coronary Artery Disease Improved Prediction by the COME-CCT Pretest Probability Calculator With Cardiac CT
Source: JACC Adv. 2025 Jul 28;4(8):102014. doi: 10.1016/j.jacadv.2025.102014 (PMC12320659; doi:10.1016/j.jacadv.2025.102014)
Supplement: Supplemental Material [file mmc1.docx]

**SUPPLEMENTAL APPENDIX**

**Expanded Details of the COME-CCT CONSORTIUM**

**Data Management Team**

Robert Röhle, Mario Walther, Robert Haase, Georg M. Schuetz, Viktoria Wieske, and Marc Dewey.

**Steering Committee**

The steering committee consists of four clinical experts for computed tomography: Stephan Achenbach, Erlangen, Germany; Matthew Budoff, Los Angeles, California, USA; Mario J Garcia, New York, New York, USA; Marc Dewey, Berlin, Germany, and one clinical expert for invasive coronary angiography: Michael Laule, Berlin, Germany. The steering committee is completed by the project’s statistician Peter Schlattmann, Jena, Germany.

**Coordinating Center**

Georg M. Schuetz, Robert Röhle, Robert Haase, Michael Laule, Viktoria Wieske, Marc Dewey (principal investigator).


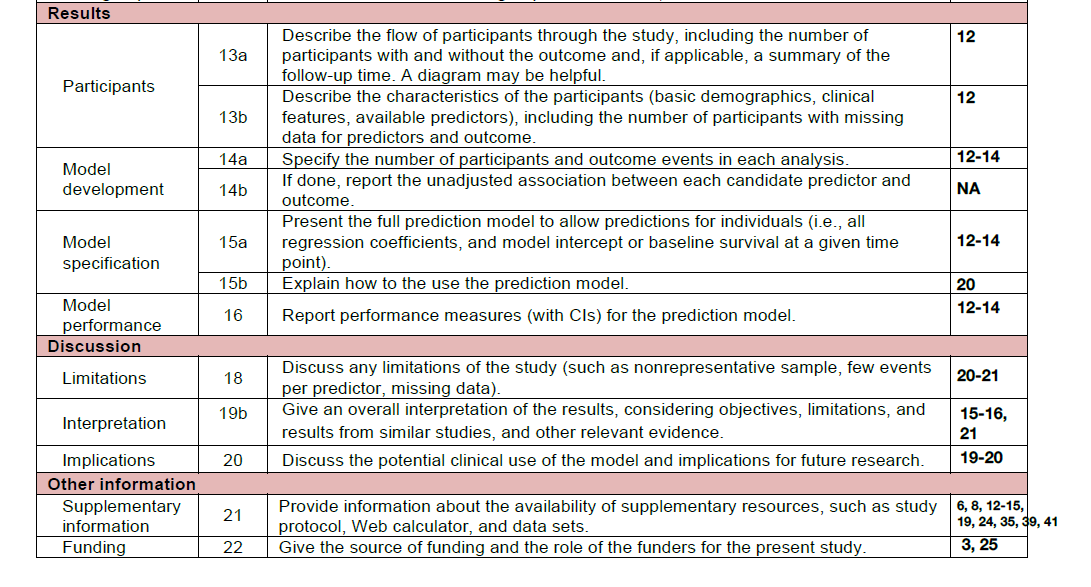

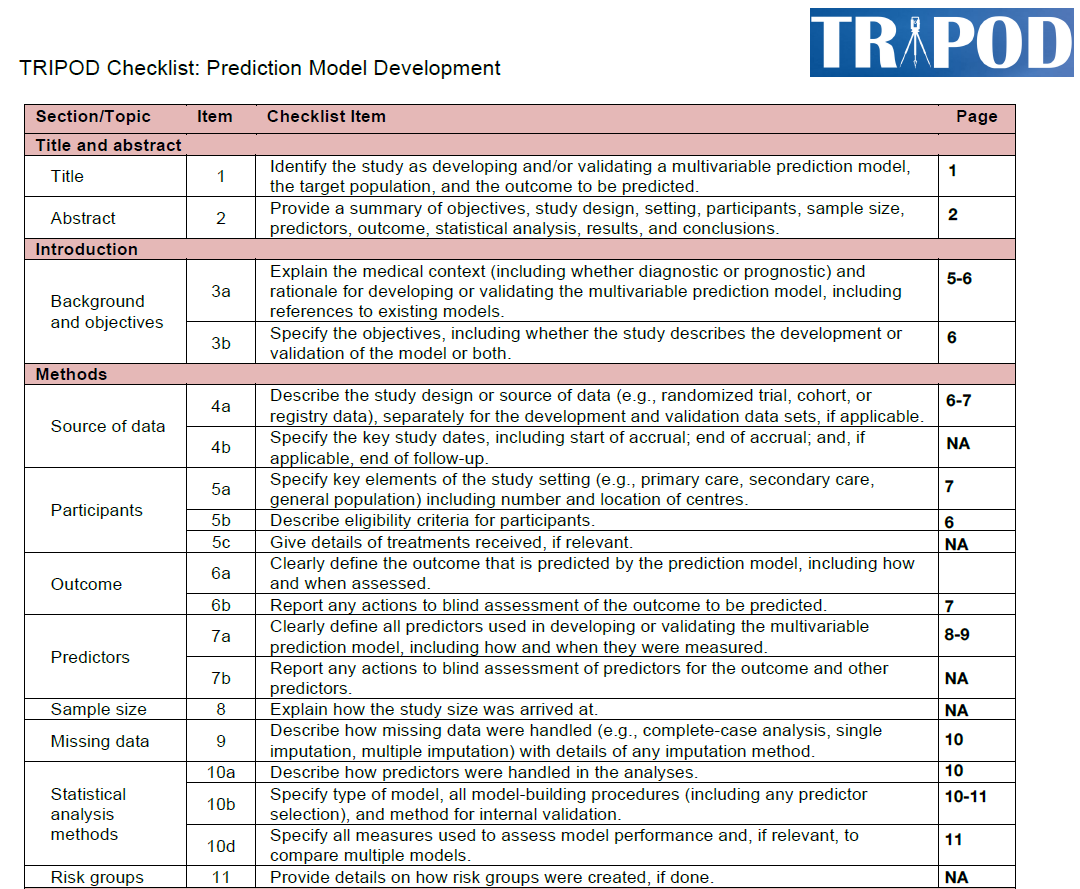
**Expanded Methods: TRIPOD Checklist**

**Supplemental Table 1 General study characteristics**

| **Study characteristics** | |
| --- | --- |
| Number of diagnostic accuracy studies* | 60 |
| Number of diagnostic accuracy datasets* | 59 |
| Publication years | 2004 - 2014 |
| Mean number of study patients per dataset (SD) | 90.4 (86.7) |
| Median number of study patients per dataset (IQR)† | 75 (36 - 108) |

IQR=interquartile range.

*The number of references of included studies and datasets vary because some datasets were referenced with more than one publication by the cooperating partners. For all statistical calculations the number of included datasets was used. Study No53 was combined with study No52 to form one study dataset since only two patients were included from study 53. Thus, 54 datasets are analyzed here.

† The total number of patients in the original studies ranged from 8 to 356 patients and the number of study patients represents the number of eligible patients per study who met the inclusion criteria for this analysis (Figure 1).

**Supplemental Table 2 Published coefficients of the updated Diamond and Forrester model^1^**

| Intercept | -4.373083 |
| --- | --- |
| Age | 0.0427672 |
| Sex (male vs. female) | 1.340049 |
| Typical angina vs. Non-specific chest pain (=Nonanginal/Other chest discomfort) | 1.905018 |
| Atypical angina vs. Non-specific chest pain  (=Nonanginal/Other chest discomfort) | 0.6353402 |

**Supplemental Table 3 Results of dataset-specific performance of the COME-CCT-PTP calculator**

| **Study ID** | **Patients** | **Patients with CAD** | **Patients without CAD** | **Study Prevalence** | **AUC** | **Brier Score** | **Maximum**  **Brier Score** | **Scaled Brier Score** | **Discrimination Slope** |
| --- | --- | --- | --- | --- | --- | --- | --- | --- | --- |
| No1 | 49 | 30 | 19 | 61.2% | 81.5% | 0.193 | 0.237 | 0.185 | 0.210 |
| No2 | 99 | 35 | 64 | 35.4% | 62.7% | 0.237 | 0.229 | -0.038 | 0.050 |
| No3 | 150 | 59 | 91 | 39.3% | 74.5% | 0.205 | 0.239 | 0.142 | 0.138 |
| No4 | 80 | 39 | 41 | 48.8% | 72.3% | 0.224 | 0.250 | 0.105 | 0.120 |
| No5 | 24 | 9 | 15 | 37.5% | 74.8% | 0.233 | 0.234 | 0.004 | 0.124 |
| No6 | 67 | 29 | 38 | 43.3% | 61.4% | 0.250 | 0.245 | -0.019 | 0.063 |
| No7 | 170 | 84 | 86 | 49.4% | 64.2% | 0.268 | 0.250 | -0.074 | 0.048 |
| No8 | 127 | 46 | 81 | 36.2% | 51.1% | 0.246 | 0.231 | -0.063 | 0.004 |
| No9 | 210 | 180 | 30 | 85.7% | 52.4% | 0.304 | 0.122 | -1.482 | 0.012 |
| No10 | 65 | 22 | 43 | 33.8% | 72.3% | 0.305 | 0.224 | -0.363 | 0.090 |
| No11 | 129 | 67 | 62 | 51.9% | 61.4% | 0.249 | 0.250 | 0.002 | 0.068 |
| No12 | 29 | 11 | 18 | 37.9% | 57.1% | 0.279 | 0.235 | -0.183 | 0.042 |
| No13 | 75 | 50 | 25 | 66.7% | 81.8% | 0.196 | 0.222 | 0.119 | 0.158 |
| No14 | 26 | 19 | 7 | 73.1% | 86.8% | 0.186 | 0.197 | 0.053 | 0.223 |
| No15 | 265 | 136 | 129 | 51.3% | 69.0% | 0.222 | 0.250 | 0.110 | 0.113 |
| No16 | 99 | 31 | 68 | 31.3% | 73.6% | 0.241 | 0.215 | -0.120 | 0.118 |
| No17 | 50 | 20 | 30 | 40.0% | 85.5% | 0.171 | 0.240 | 0.289 | 0.203 |
| No18 | 230 | 72 | 158 | 31.3% | 53.9% | 0.277 | 0.215 | -0.290 | 0.022 |
| No19 | 8 | 3 | 5 | 37.5% | 73.3% | 0.236 | 0.234 | -0.006 | 0.122 |
| No20 | 41 | 24 | 17 | 58.5% | 86.8% | 0.161 | 0.243 | 0.337 | 0.247 |
| No21 | 75 | 17 | 58 | 22.7% | 64.9% | 0.196 | 0.175 | -0.118 | 0.098 |
| **Study ID** | **Patients** | **Patients with CAD** | **Patients without CAD** | **Study Prevalence** | **AUC** | **Brier Score** | **Maximum**  **Brier Score** | **Scaled Brier Score** | **Discrimination Slope** |
| No22 | 15 | 5 | 10 | 33.3% | 84.0% | 0.168 | 0.222 | 0.242 | 0.179 |
| No23 | 32 | 15 | 17 | 46.9% | 70.8% | 0.232 | 0.249 | 0.068 | 0.110 |
| No24 | 31 | 20 | 11 | 64.5% | 60.5% | 0.215 | 0.229 | 0.060 | 0.060 |
| No25 | 574 | 274 | 300 | 47.7% | 74.4% | 0.208 | 0.249 | 0.167 | 0.143 |
| No26 | 42 | 22 | 20 | 52.4% | 78.6% | 0.251 | 0.249 | -0.005 | 0.082 |
| No27 | 83 | 39 | 44 | 47.0% | 76.7% | 0.201 | 0.249 | 0.191 | 0.150 |
| No28 | 88 | 44 | 44 | 50.0% | 55.7% | 0.257 | 0.250 | -0.029 | 0.041 |
| No29 | 243 | 101 | 142 | 41.6% | 64.1% | 0.225 | 0.243 | 0.072 | 0.080 |
| No30 | 32 | 6 | 26 | 18.8% | 80.8% | 0.166 | 0.152 | -0.089 | 0.131 |
| No31 | 99 | 38 | 61 | 38.4% | 86.1% | 0.166 | 0.237 | 0.297 | 0.242 |
| No32 | 22 | 12 | 10 | 54.5% | 75.0% | 0.224 | 0.248 | 0.095 | 0.151 |
| No33 | 36 | 20 | 16 | 55.6% | 75.8% | 0.209 | 0.247 | 0.156 | 0.156 |
| No34 | 61 | 33 | 28 | 54.1% | 47.8% | 0.268 | 0.248 | -0.080 | 0.019 |
| No35 | 99 | 43 | 56 | 43.4% | 77.2% | 0.206 | 0.246 | 0.162 | 0.149 |
| No36 | 13 | 2 | 11 | 15.4% | 95.5% | 0.165 | 0.130 | -0.266 | 0.282 |
| No37 | 68 | 26 | 42 | 38.2% | 69.6% | 0.212 | 0.236 | 0.100 | 0.080 |
| No38 | 64 | 43 | 21 | 67.2% | 52.5% | 0.263 | 0.220 | -0.195 | 0.010 |
| No39 | 178 | 93 | 85 | 52.2% | 80.1% | 0.191 | 0.249 | 0.233 | 0.180 |
| No40 | 108 | 69 | 39 | 63.9% | 62.2% | 0.236 | 0.231 | -0.024 | 0.070 |
| No41 | 81 | 18 | 63 | 22.2% | 93.5% | 0.156 | 0.173 | 0.097 | 0.281 |
| No42 | 33 | 14 | 19 | 42.4% | 60.9% | 0.241 | 0.244 | 0.015 | 0.033 |
| No43 | 60 | 5 | 55 | 8.3% | 52.2% | 0.159 | 0.076 | -1.083 | 0.007 |
| No44 | 33 | 21 | 12 | 63.6% | 77.4% | 0.194 | 0.231 | 0.162 | 0.115 |
| No45 | 100 | 29 | 71 | 29.0% | 76.9% | 0.184 | 0.206 | 0.105 | 0.159 |
| **Study ID** | **Patients** | **Patients with CAD** | **Patients without CAD** | **Study Prevalence** | **AUC** | **Brier Score** | **Maximum**  **Brier Score** | **Scaled Brier Score** | **Discrimination Slope** |
| No46 | 110 | 44 | 66 | 40.0% | 81.9% | 0.195 | 0.240 | 0.190 | 0.191 |
| No47 | 116 | 63 | 53 | 54.3% | 64.9% | 0.245 | 0.248 | 0.013 | 0.061 |
| No48 | 144 | 95 | 49 | 66.0% | 69.5% | 0.220 | 0.224 | 0.021 | 0.107 |
| No49 | 96 | 90 | 6 | 93.8% | 62.8% | 0.258 | 0.059 | -3.403 | 0.085 |
| No50 | 39 | 11 | 28 | 28.2% | 51.5% | 0.240 | 0.202 | -0.184 | 0.005 |
| No51 | 76 | 40 | 36 | 52.6% | 75.6% | 0.208 | 0.249 | 0.164 | 0.137 |
| No52 | 107 | 59 | 48 | 55.1% | 76.9% | 0.198 | 0.247 | 0.198 | 0.160 |
| No53 | 98 | 45 | 53 | 45.9% | 79.4% | 0.198 | 0.248 | 0.201 | 0.174 |
| No54 | 20 | 11 | 9 | 55.0% | 64.6% | 0.229 | 0.248 | 0.073 | 0.083 |
| No55 | 113 | 90 | 23 | 79.6% | 53.2% | 0.332 | 0.162 | -1.049 | 0.022 |
| No56 | 37 | 27 | 10 | 73.0% | 62.4% | 0.305 | 0.197 | -0.547 | 0.066 |
| No57+58^*^ | 16 | 5 | 11 | 31.3% | 87.3% | 0.179 | 0.215 | 0.167 | 0.243 |
| No59 | 60 | 8 | 52 | 13.3% | 88.7% | 0.109 | 0.116 | 0.057 | 0.203 |
| No60 | 37 | 10 | 27 | 27.0% | 85.6% | 0.156 | 0.197 | 0.210 | 0.176 |
|  |  |  |  |  |  |  |  |  |  |
| **Total** | **5332** | **2573** | **2759** | **48.3%** | **67.9%** | **0.226** | **0.250** | **0.097** | **0.107** |

CAD = coronary artery disease; AUC = area under the receiver-operating-characteristic curve.

Median AUC=73.3% (IQR: 62.3% to 79.7%); Median Brier-Score = 0.220 (IQR: 0.194 to 0.245); Median Max Brier-Score = 0.235 (IQR: 0.215 to 0.248); Median Scaled Brier-Score= 0.057 (IQR: -0.077 to 0.162); Median Discrimination Slope = 0.115 (IQR: 0.062 to 0.160).

***** For the logistic regression modelling procedure study No57 was combined with study No58 to one study since only two patients were included from study No58 for this cohort.

**Supplemental Table 4 Internal validation of the COME-CCT-PTP calculator, CTA alone and Combined COME-CCT-PTP calculator with CTA***

| **Performance Measure** | **Original  Model** | **Bootstrap  Sample** | **Original  Sample** | **Optimism** | **Bootstrap  corrected** |  |
| --- | --- | --- | --- | --- | --- | --- |
| **COME-CCT-PTP calculator** | | | | | |  |
| AUC† | 67.9% | 68.0% | 67.8% | 0.160 | 67.7% |  |
| Brier Score‡ | 0.226 | 0.225 | 0.226 | -0.001 | 0.226 |  |
| Scaled Brier Score§ | 0.097 | 0.098 | 0.095 | 0.002 | 0.094 |  |
| Discrimination Slope\|\| | 0.107 | 0.107 | 0.106 | 0.001 | 0.105 |  |
| Calibration Slope# | 0.905 | 0.910 | 0.902 | 0.008 | 0.897 |  |
| Accuracy¶ | 62.5% | 63.0% | 62.7% | 0.282 | 62.2% |  |
| Misclassification** | 37.5% | 37.0% | 37.3% | -0.282 | 37.8% |  |
| **CTA alone** | | | | | |  |
| AUC† | 80.5% | 80.5% | 80.5% | -0.020 | 80.6% |  |
| Brier Score‡ | 0.156 | 0.156 | 0.156 | 0.000 | 0.156 |  |
| Scaled Brier Score§ | 0.377 | 0.376 | 0.376 | 0.000 | 0.377 |  |
| Discrimination Slope\|\| | 0.359 | 0.356 | 0.356 | 0.000 | 0.359 |  |
| Calibration Slope# | 1.069 | 1.080 | 1.080 | 0.000 | 1.070 |  |
| Accuracy¶ | 80.3% | 80.3% | 80.3% | -0.019 | 80.3% |  |
| Misclassification** | 19.7% | 19.7% | 19.7% | 0.019 | 19.7% |  |
| **Combined COME-CCT-PTP calculator and CTA** | | | | | |  |
| AUC† | 86.0% | 86.0% | 86.0% | 0.017 | 86.0% |  |
| Brier Score‡ | 0.146 | 0.147 | 0.147 | 0.000 | 0.147 |  |
| Scaled Brier Score§ | 0.414 | 0.413 | 0.412 | 0.001 | 0.413 |  |
| Discrimination Slope\|\| | 0.400 | 0.397 | 0.396 | 0.000 | 0.399 |  |
| Calibration Slope# | 1.065 | 1.076 | 1.074 | 0.001 | 1.064 |  |
| Accuracy¶ | 80.5% | 80.6% | 80.4% | 0.125 | 80.4% |  |
| Misclassification** | 19.5% | 19.4% | 19.6% | -0.125 | 19.6% |  |

AUC=area under the receiver-operating-characteristic curve. *Internal validation of pretest probability calculation using Harrell’s approach based on 250 bootstrap samples with the following columns: *original model* reflects the performance measures based on original sample and prediction model; *bootstrap sample* represents the averaged performance measures based on results of 250 bootstrap samples applying prediction model with the same covariates and random effects as for original sample; *original sample* includes the averaged performance measures based on original sample using prediction model generated by each of the 250 bootstrap samples; *optimism* as a measure of biasedness is defined as difference of performance measures resulting from the bootstrap sample and original sample; *bootstrap corrected* represents difference between performance of original model and the optimism, where this is a nearly unbiased estimate of the expected values of the optimism that would be obtained in external validation. †AUC as a concordance statistic. ‡Brier score measuring the accuracy of probabilistic predictions, i.e., the average of the squared differences between actual outcome and predicted probabilities (prediction error). Brier score of 0 indicates a perfect model. ‡ Scaled Brier score is scaled by its maximum score, taking the incidence of the outcome into account. A scaled score of 1 indicates a perfect model. ||Discrimination slope=Difference in mean of predicted probabilities between outcomes #Calibration slope=measure of agreement between actual outcome and predicted probability ¶Accuracy, i.e., the fraction of correct classification based on Youden index and its associated optimal cut-off for predicted probabilities. **Misclassification = 100% - accurac

**Supplemental Table 5 Internal-external cross-validation of dataset-specific performance of the COME-CCT-PTP calculator**

| **Study ID** | **Patients** | **Calibration**  **Slope** | **AUC** | **Exp./Obs.** |
| --- | --- | --- | --- | --- |
| No1 | 49 | 1.858 | 81.0% | 0.744 |
| No2 | 99 | 0.533 | 62.3% | 1.253 |
| No3 | 150 | 1.381 | 74.9% | 1.180 |
| No4 | 80 | 1.062 | 71.8% | 0.811 |
| No5 | 24 | 1.130 | 71.9% | 1.476 |
| No6 | 67 | 0.484 | 62.3% | 1.157 |
| No7 | 170 | 0.980 | 61.3% | 0.654 |
| No8 | 127 | -0.118 | 51.0% | 0.821 |
| No9 | 210 | 0.076 | 51.3% | 0.510 |
| No10 | 65 | 1.674 | 72.3% | 1.979 |
| No11 | 129 | 0.585 | 60.9% | 0.813 |
| No12 | 29 | -0.448 | 57.6% | 0.949 |
| No13 | 75 | 2.430 | 82.4% | 0.768 |
| No14 | 26 | 2.570 | 86.1% | 0.693 |
| No15 | 265 | 1.073 | 68.9% | 0.896 |
| No16 | 99 | 1.318 | 73.3% | 1.758 |
| No17 | 50 | 2.168 | 83.7% | 1.055 |
| No18 | 230 | 0.174 | 53.8% | 1.681 |
| No19 | 8 | 1.871 | 73.3% | 1.507 |
| No20 | 41 | 2.122 | 86.3% | 0.924 |
| No21 | 75 | 0.950 | 65.3% | 1.789 |
| No22 | 15 | 2.620 | 84.0% | 1.191 |
| No23 | 32 | 0.819 | 70.8% | 1.233 |
| No24 | 31 | 0.931 | 60.5% | 1.053 |
| No25 | 574 | 1.407 | 73.7% | 1.089 |
| No26 | 42 | 3.089 | 78.6% | 0.634 |
| No27 | 83 | 1.625 | 76.5% | 1.081 |
| No28 | 88 | 0.354 | 55.1% | 0.923 |
| No29 | 243 | 0.864 | 63.8% | 1.016 |
| No30 | 32 | 1.578 | 80.8% | 2.012 |
| No31 | 99 | 1.965 | 85.7% | 1.203 |
| No32 | 22 | 0.881 | 74.2% | 0.760 |
| No33 | 36 | 1.670 | 75.8% | 0.779 |
| No34 | 61 | 0.151 | 47.9% | 0.933 |
| No35 | 99 | 1.629 | 77.0% | 1.202 |
| No36 | 13 | 4.493 | 95.5% | 2.919 |
| No37 | 68 | 1.483 | 69.6% | 1.047 |
| No38 | 64 | -0.147 | 52.8% | 0.799 |
| No39 | 178 | 1.625 | 80.4% | 0.926 |
| No40 | 108 | 0.687 | 62.3% | 0.806 |
| No41 | 81 | 4.432 | 93.4% | 2.068 |
| No42 | 33 | 0.422 | 57.5% | 0.966 |
| No43 | 60 | -0.192 | 49.6% | 4.224 |
| No44 | 33 | 2.166 | 77.4% | 1.032 |
| No45 | 100 | 1.537 | 77.4% | 1.434 |
| No46 | 110 | 2.007 | 81.9% | 1.332 |
| No47 | 116 | 1.188 | 66.2% | 0.754 |
| No48 | 144 | 1.127 | 69.5% | 0.783 |
| No49 | 96 | 1.294 | 63.8% | 0.524 |
| No50 | 39 | -0.031 | 51.6% | 1.464 |
| No51 | 76 | 1.699 | 75.7% | 0.861 |
| No52 | 107 | 1.426 | 76.6% | 1.046 |
| No53 | 98 | 1.693 | 79.3% | 1.169 |
| No54 | 20 | 1.206 | 64.6% | 1.161 |
| No55^#^ | 113 | 0.433 | 54.1% | 0.457 |
| No56 | 37 | 0.822 | 61.5% | 0.512 |
| No57+58^*^ | 16 | 2.084 | 87.3% | 1.578 |
| No59 | 60 | 3.307 | 88.3% | 2.222 |
| No60 | 37 | 2.199 | 85.4% | 1.293 |
|  |  |  |  |  |
| **Total** | **5332** | **0.889** | **67.8%** | **0.965** |

AUC = area under the receiver-operating-characteristic curve; Exp./Obs. = Ratio of expected (or predicted) and observed outcomes.

Median AUC = 72.3 (IQR: 61.9% to 79.9%); Average calibration slope of 1.13 (95% CI: 0.94 to 1.32) based on a random-effects meta-analysis; Median ratio of expected/observed = 1.047 (IQR: 0.809 to 1.313).

***** For the logistic regression modelling procedure study No57 was combined with study No58 to one study since only two patients were included from study No58 for this cohort.

^#^ The logistic regression model with dataset-specific random intercept failed to converge in R for these studies.

**Supplemental Table 6 Sensitivity analysis. Use of average intercepts according to Debray et al (2013).^2^ Internal-external cross-validation of dataset-specific performance of the COME-CCT-PTP calculator**

| **Study ID** | **Patients** | **Calibration**  **Slope** | **AUC** | **Exp./Obs.** |
| --- | --- | --- | --- | --- |
| No1 | 49 | 1.638 | 81.0% | 0.739 |
| No2 | 99 | 0.467 | 62.3% | 1.235 |
| No3 | 150 | 1.213 | 74.9% | 1.171 |
| No4 | 80 | 0.937 | 71.8% | 0.791 |
| No5 | 24 | 1.004 | 71.9% | 1.491 |
| No6 | 67 | 0.426 | 62.3% | 1.158 |
| No7 | 170 | 0.870 | 61.3% | 0.615 |
| No8 | 127 | -0.104 | 51.0% | 0.763 |
| No9 | 210 | 0.070 | 51.3% | 0.502 |
| No10 | 65 | 1.492 | 72.3% | 2.032 |
| No11 | 129 | 0.513 | 60.9% | 0.799 |
| No12 | 29 | -0.396 | 57.6% | 0.911 |
| No13 | 75 | 2.129 | 82.4% | 0.771 |
| No14 | 26 | 2.258 | 86.1% | 0.696 |
| No15 | 265 | 0.941 | 68.9% | 0.889 |
| No16 | 99 | 1.164 | 73.3% | 1.778 |
| No17 | 50 | 1.897 | 83.7% | 1.035 |
| No18 | 230 | 0.152 | 53.8% | 1.693 |
| No19 | 8 | 1.645 | 73.3% | 1.533 |
| No20 | 41 | 1.860 | 86.3% | 0.933 |
| No21 | 75 | 0.836 | 65.3% | 1.744 |
| No22 | 15 | 2.291 | 84.0% | 1.159 |
| No23 | 32 | 0.819 | 70.8% | 1.233 |
| No24 | 31 | 0.830 | 60.5% | 1.084 |
| No25 | 574 | 1.233 | 73.7% | 1.096 |
| No26 | 42 | 2.733 | 78.6% | 0.598 |
| No27 | 83 | 1.423 | 76.5% | 1.085 |
| No28 | 88 | 0.308 | 55.1% | 0.916 |
| No29 | 243 | 0.755 | 63.8% | 0.996 |
| No30 | 32 | 1.394 | 80.8% | 1.941 |
| No31 | 99 | 1.724 | 85.7% | 1.197 |
| No32 | 22 | 0.792 | 74.2% | 0.746 |
| No33 | 36 | 1.473 | 75.8% | 0.767 |
| No34 | 61 | 0.133 | 47.9% | 0.936 |
| No35 | 99 | 1.428 | 77.0% | 1.210 |
| No36 | 13 | 3.955 | 95.5% | 2.878 |
| No37 | 68 | 1.303 | 69.6% | 1.019 |
| No38 | 64 | -0.127 | 52.8% | 0.807 |
| No39 | 178 | 1.430 | 80.4% | 0.925 |
| No40 | 108 | 0.601 | 62.3% | 0.810 |
| No41 | 81 | 3.906 | 93.4% | 2.052 |
| No42 | 33 | 0.375 | 57.5% | 0.943 |
| No43 | 60 | -0.165 | 49.6% | 4.021 |
| No44 | 33 | 1.912 | 77.4% | 1.061 |
| No45 | 100 | 1.349 | 77.4% | 1.405 |
| No46 | 110 | 1.761 | 81.9% | 1.342 |
| No47 | 116 | 1.042 | 66.2% | 0.734 |
| No48 | 144 | 0.990 | 69.5% | 0.787 |
| No49 | 96 | 1.157 | 63.8% | 0.523 |
| No50 | 39 | -0.029 | 51.6% | 1.425 |
| No51 | 76 | 1.491 | 75.7% | 0.852 |
| No52 | 107 | 1.254 | 76.6% | 1.062 |
| No53 | 98 | 1.487 | 79.3% | 1.181 |
| No54 | 20 | 1.066 | 64.6% | 1.190 |
| No55^#^ | 113 | 0.385 | 54.1% | 0.441 |
| No56 | 37 | 0.727 | 61.5% | 0.494 |
| No57+58^*^ | 16 | 1.827 | 87.3% | 1.576 |
| No59 | 60 | 2.912 | 88.3% | 2.071 |
| No60 | 37 | 1.936 | 85.4% | 1.238 |
|  |  |  |  |  |
| **Total** | **5332** | **0.782** | **67.8%** | **0.959** |

AUC = area under the receiver-operating-characteristic curve; Exp./Obs. = Ratio of expected (or predicted) and observed outcomes.

Median AUC = 72.3% (IQR: 61.9% to 79.9); Average calibration slope of 0.996 (95% CI: 0.83 to 1.16) based on a random-effects meta-analysis; Median ratio of expected/observed 1.061 (IQR: 0.795 to 1.290).

***** For the logistic regression modelling procedure study No57 was combined with study No58 to one study since only two patients were included from study No58 for this cohort.

^#^ The multivariable logistic regression model with dataset-specific random intercept failed to converge in R for these studies.

**Supplemental Table 7 Results of dataset-specific performance of the prediction model including CTA alone**

| **Study ID** | **Patients** | **Patients With CAD** | **Patients Without CAD** | **Study  Prevalence** | **AUC** | **Brier Score** | **Maximum Brier Score** | **Scaled  Brier Score** | **Discrimination Slope** |
| --- | --- | --- | --- | --- | --- | --- | --- | --- | --- |
| No1 | 49 | 30 | 19 | 61.2% | 100.0% | 0.052 | 0.237 | 0.780 | 0.587 |
| No2 | 99 | 35 | 64 | 35.4% | 93.2% | 0.078 | 0.229 | 0.657 | 0.508 |
| No3 | 150 | 59 | 91 | 39.3% | 91.7% | 0.091 | 0.239 | 0.619 | 0.490 |
| No4 | 80 | 39 | 41 | 48.8% | 91.5% | 0.091 | 0.250 | 0.636 | 0.487 |
| No5 | 24 | 9 | 15 | 37.5% | 94.4% | 0.068 | 0.234 | 0.712 | 0.522 |
| No6 | 67 | 29 | 38 | 43.3% | 88.7% | 0.109 | 0.245 | 0.557 | 0.454 |
| No7 | 170 | 84 | 86 | 49.4% | 93.6% | 0.079 | 0.250 | 0.682 | 0.512 |
| No8 | 127 | 46 | 81 | 36.2% | 93.8% | 0.080 | 0.231 | 0.654 | 0.514 |
| No9 | 210 | 180 | 30 | 85.7% | 68.9% | 0.138 | 0.122 | -0.124 | 0.222 |
| No10 | 65 | 22 | 43 | 33.8% | 50.9% | 0.282 | 0.224 | -0.259 | 0.011 |
| No11 | 129 | 67 | 62 | 51.9% | 83.4% | 0.137 | 0.250 | 0.452 | 0.392 |
| No12 | 29 | 11 | 18 | 37.9% | 97.2% | 0.058 | 0.235 | 0.753 | 0.554 |
| No13 | 75 | 50 | 25 | 66.7% | 86.0% | 0.124 | 0.222 | 0.440 | 0.423 |
| No14 | 26 | 19 | 7 | 73.1% | 97.4% | 0.084 | 0.197 | 0.575 | 0.556 |
| No15 | 265 | 136 | 129 | 51.3% | 85.5% | 0.127 | 0.250 | 0.492 | 0.417 |
| No16 | 99 | 31 | 68 | 31.3% | 84.6% | 0.122 | 0.215 | 0.432 | 0.406 |
| No17 | 50 | 20 | 30 | 40.0% | 40.0% | 0.394 | 0.240 | -0.641 | 0.117 |
| No18 | 230 | 72 | 158 | 31.3% | 66.4% | 0.242 | 0.215 | -0.124 | 0.193 |
| No19 | 8 | 3 | 5 | 37.5% | 100.0% | 0.040 | 0.234 | 0.829 | 0.587 |
| No20 | 41 | 24 | 17 | 58.5% | 78.2% | 0.158 | 0.243 | 0.348 | 0.331 |
| No21 | 75 | 17 | 58 | 22.7% | 85.5% | 0.119 | 0.175 | 0.323 | 0.417 |
| No22 | 15 | 5 | 10 | 33.3% | 100.0% | 0.038 | 0.222 | 0.829 | 0.587 |
| No23 | 32 | 15 | 17 | 46.9% | 94.1% | 0.077 | 0.249 | 0.691 | 0.518 |
| No24 | 31 | 20 | 11 | 64.5% | 95.5% | 0.070 | 0.229 | 0.692 | 0.534 |
| No25 | 574 | 274 | 300 | 47.7% | 49.0% | 0.344 | 0.249 | -0.379 | 0.012 |
| No26 | 42 | 22 | 20 | 52.4% | 82.7% | 0.137 | 0.249 | 0.451 | 0.384 |
| No27 | 83 | 39 | 44 | 47.0% | 80.9% | 0.155 | 0.249 | 0.379 | 0.363 |
| No28 | 88 | 44 | 44 | 50.0% | 83.0% | 0.141 | 0.250 | 0.437 | 0.387 |
| No29 | 243 | 101 | 142 | 41.6% | 86.8% | 0.121 | 0.243 | 0.501 | 0.432 |
| No30 | 32 | 6 | 26 | 18.8% | 85.9% | 0.099 | 0.152 | 0.347 | 0.421 |
| No31 | 99 | 38 | 61 | 38.4% | 59.3% | 0.280 | 0.237 | -0.185 | 0.109 |
| No32 | 22 | 12 | 10 | 54.5% | 80.0% | 0.142 | 0.248 | 0.426 | 0.352 |
| No33 | 36 | 20 | 16 | 55.6% | 93.8% | 0.078 | 0.247 | 0.685 | 0.514 |
| No34 | 61 | 33 | 28 | 54.1% | 91.1% | 0.091 | 0.248 | 0.635 | 0.482 |
| No35 | 99 | 43 | 56 | 43.4% | 93.2% | 0.082 | 0.246 | 0.665 | 0.507 |
| No36 | 13 | 2 | 11 | 15.4% | 95.5% | 0.068 | 0.130 | 0.474 | 0.534 |
| No37 | 68 | 26 | 42 | 38.2% | 73.1% | 0.209 | 0.236 | 0.115 | 0.271 |
| No38 | 64 | 43 | 21 | 67.2% | 78.7% | 0.165 | 0.220 | 0.251 | 0.337 |
| No39 | 178 | 93 | 85 | 52.2% | 92.5% | 0.087 | 0.249 | 0.652 | 0.498 |
| No40 | 108 | 69 | 39 | 63.9% | 74.9% | 0.150 | 0.231 | 0.349 | 0.292 |
| No41 | 81 | 18 | 63 | 22.2% | 93.3% | 0.072 | 0.173 | 0.582 | 0.508 |
| No42 | 33 | 14 | 19 | 42.4% | 63.9% | 0.254 | 0.244 | -0.040 | 0.163 |
| No43 | 60 | 5 | 55 | 8.3% | 72.7% | 0.199 | 0.076 | -1.608 | 0.267 |
| No44 | 33 | 21 | 12 | 63.6% | 65.5% | 0.227 | 0.231 | 0.020 | 0.182 |
| No45 | 100 | 29 | 71 | 29.0% | 85.0% | 0.128 | 0.206 | 0.380 | 0.410 |
| No46 | 110 | 44 | 66 | 40.0% | 89.8% | 0.104 | 0.240 | 0.569 | 0.467 |
| No47 | 116 | 63 | 53 | 54.3% | 82.1% | 0.133 | 0.248 | 0.464 | 0.377 |
| No48 | 144 | 95 | 49 | 66.0% | 80.0% | 0.138 | 0.224 | 0.384 | 0.352 |
| No49 | 96 | 90 | 6 | 93.8% | 82.8% | 0.086 | 0.059 | -0.472 | 0.385 |
| No50 | 39 | 11 | 28 | 28.2% | 88.3% | 0.105 | 0.202 | 0.481 | 0.450 |
| No51 | 76 | 40 | 36 | 52.6% | 91.7% | 0.088 | 0.249 | 0.645 | 0.489 |
| No52 | 107 | 59 | 48 | 55.1% | 71.7% | 0.214 | 0.247 | 0.136 | 0.254 |
| No53 | 98 | 45 | 53 | 45.9% | 86.1% | 0.127 | 0.248 | 0.487 | 0.423 |
| No54 | 20 | 11 | 9 | 55.0% | 74.2% | 0.192 | 0.248 | 0.224 | 0.285 |
| No55 | 113 | 90 | 23 | 79.6% | 80.4% | 0.121 | 0.162 | 0.252 | 0.357 |
| No56 | 37 | 27 | 10 | 73.0% | 80.0% | 0.114 | 0.197 | 0.423 | 0.352 |
| No57+58^*^ | 16 | 5 | 11 | 31.3% | 86.4% | 0.133 | 0.215 | 0.379 | 0.427 |
| No59 | 60 | 8 | 52 | 13.3% | 94.2% | 0.079 | 0.116 | 0.314 | 0.519 |
| No60 | 37 | 10 | 27 | 27.0% | 87.6% | 0.108 | 0.197 | 0.451 | 0.441 |
|  |  |  |  |  |  |  |  |  |  |
| **Total** | **5332** | **2573** | **2759** | **48.3%** | **80.5%** | **0.156** | **0.250** | **0.377** | **0.359** |

CAD = coronary artery disease; AUC = area under the receiver-operating-characteristic curve.

Median AUC=85.9% (IQR: 79.4% to 93.2%); Median Brier-Score = 0.121 (IQR: 0.083 to 0.146); Median Max Brier-Score = 0.235 (IQR: 0.215 to 0.248); Median Scaled Brier-Score= 0.451 (IQR: 0.283 to 0.636); Median Discrimination Slope = 0.421 (IQR: 0.345 to 0.507).

***** For the logistic regression modelling procedure study No57 was combined with study No58 to one study since only two patients were included from study No58 for this cohort.

**Supplemental Table 8 Internal-external cross-validation of dataset-specific performance of the prediction model including CTA alone**

| **Study ID** | **Patients** | **Calibration Slope** | **AUC** | **Exp./Obs.** |
| --- | --- | --- | --- | --- |
| No1 | 49 | 19.292 | 100.0% | 0.820 |
| No2 | 99 | 1.922 | 93.2% | 1.051 |
| No3 | 150 | 1.917 | 91.7% | 1.058 |
| No4 | 80 | 7.744 | 91.5% | 0.989 |
| No5 | 24 | 8.411 | 94.4% | 0.908 |
| No6 | 67 | 1.549 | 88.7% | 1.003 |
| No7 | 170 | 8.337 | 93.6% | 0.956 |
| No8 | 127 | 8.084 | 93.8% | 1.119 |
| No9 | 210 | 0.915 | 68.9% | 0.775 |
| No10 | 65 | 0.037 | 50.9% | 0.792 |
| No11 | 129 | 1.295 | 83.4% | 0.961 |
| No12 | 29 | 8.311 | 97.2% | 1.024 |
| No13 | 75 | 1.382 | 86.0% | 0.812 |
| No14 | 26 | 8.477 | 97.4% | 0.751 |
| No15 | 265 | 1.374 | 85.5% | 0.937 |
| No16 | 99 | 1.231 | 84.6% | 1.148 |
| No17 | 50 | -0.347 | 40.0% | 1.429 |
| No18 | 230 | 0.511 | 66.4% | 1.509 |
| No19 | 8 | 17.718 | 100.0% | 0.974 |
| No20 | 41 | 1.083 | 78.2% | 0.931 |
| No21 | 75 | 1.292 | 85.5% | 1.513 |
| No22 | 15 | 18.467 | 100.0% | 1.023 |
| No23 | 32 | 8.172 | 94.1% | 0.974 |
| No24 | 31 | 8.522 | 95.5% | 0.838 |
| No25 | 574 | -0.023 | 49.0% | 0.868 |
| No26 | 42 | 1.405 | 82.7% | 0.997 |
| No27 | 83 | 1.055 | 80.9% | 0.955 |
| No28 | 88 | 1.229 | 83.0% | 0.970 |
| No29 | 243 | 2.088 | 86.8% | 1.142 |
| No30 | 32 | 1.314 | 85.9% | 1.565 |
| No31 | 99 | 0.283 | 59.3% | 1.296 |
| No32 | 22 | 7.094 | 80.0% | 1.049 |
| No33 | 36 | 8.280 | 93.8% | 0.905 |
| No34 | 61 | 7.788 | 91.1% | 0.942 |
| No35 | 99 | 1.949 | 93.2% | 0.962 |
| No36 | 13 | 8.022 | 95.5% | 1.831 |
| No37 | 68 | 1.156 | 73.1% | 1.426 |
| No38 | 64 | 0.982 | 78.7% | 0.799 |
| No39 | 178 | 2.108 | 92.5% | 0.919 |
| No40 | 108 | 1.546 | 74.9% | 0.966 |
| No41 | 81 | 1.917 | 93.3% | 1.381 |
| No42 | 33 | 0.528 | 63.9% | 1.311 |
| No43 | 60 | 0.718 | 72.7% | 4.497 |
| No44 | 33 | 0.519 | 65.5% | 0.869 |
| No45 | 100 | 1.287 | 85.0% | 1.316 |
| No46 | 110 | 1.917 | 89.8% | 1.099 |
| No47 | 116 | 7.546 | 82.1% | 1.030 |
| No48 | 144 | 1.275 | 80.0% | 0.878 |
| No49 | 96 | 1.856 | 82.8% | 0.743 |
| No50 | 39 | 1.478 | 88.3% | 1.266 |
| No51 | 76 | 7.808 | 91.7% | 0.949 |
| No52 | 107 | 0.664 | 71.7% | 0.847 |
| No53 | 98 | 1.472 | 86.1% | 0.809 |
| No54 | 20 | 0.791 | 74.2% | 0.903 |
| No55 | 113 | 1.332 | 80.4% | 0.790 |
| No56 | 37 | 7.395 | 80.0% | 0.870 |
| No57+58^*^ | 16 | 7.233 | 86.4% | 1.407 |
| No59 | 60 | 7.515 | 94.2% | 2.137 |
| No60 | 37 | 1.424 | 87.6% | 1.305 |
|  |  |  |  |  |
| **Total** | **5332** | **1.069** | **80.5%** | **0.980** |

AUC = area under the receiver-operating-characteristic curve; Exp./Obs. = Ratio of expected (or predicted) and observed outcomes.

Median AUC = 85.9 (IQR: 79.4% to 93.2%); Average calibration slope of 1.16 (95% CI: 0.93 to 1.40) based on a random-effects meta-analysis; Median ratio of expected/observed = 0.974 (IQR: 0.890 to 1.207).

***** For the logistic regression modelling procedure study No57 was combined with study No58 to one study since only two patients were included from study No58 for this cohort.

**Supplemental Table 9 Sensitivity analysis. Use of average intercepts according to Debray et al (2013).^2^ Internal-external cross-validation of study-specific performance of the prediction model for CTA alone**

| **Study ID** | **Patients** | **Calibration Slope** | **AUC** | **Exp./Obs.** |
| --- | --- | --- | --- | --- |
| No1 | 49 | 18.033 | 100.0% | 0.826 |
| No2 | 99 | 1.793 | 93.2% | 1.041 |
| No3 | 150 | 1.789 | 91.7% | 1.055 |
| No4 | 80 | 7.227 | 91.5% | 0.994 |
| No5 | 24 | 7.854 | 94.4% | 0.895 |
| No6 | 67 | 1.446 | 88.7% | 1.003 |
| No7 | 170 | 7.781 | 93.6% | 0.960 |
| No8 | 127 | 7.547 | 93.8% | 1.114 |
| No9 | 210 | 0.861 | 68.9% | 0.787 |
| No10 | 65 | 0.035 | 50.9% | 0.769 |
| No11 | 129 | 1.208 | 83.4% | 0.967 |
| No12 | 29 | 7.759 | 97.2% | 1.017 |
| No13 | 75 | 1.293 | 86.0% | 0.820 |
| No14 | 26 | 7.935 | 97.4% | 0.758 |
| No15 | 265 | 1.282 | 85.5% | 0.941 |
| No16 | 99 | 1.149 | 84.6% | 1.136 |
| No17 | 50 | -0.325 | 40.0% | 1.445 |
| No18 | 230 | 0.479 | 66.4% | 1.515 |
| No19 | 8 | 16.551 | 100.0% | 0.964 |
| No20 | 41 | 1.011 | 78.2% | 0.940 |
| No21 | 75 | 1.208 | 85.5% | 1.492 |
| No22 | 15 | 17.246 | 100.0% | 1.009 |
| No23 | 32 | 7.629 | 94.1% | 0.976 |
| No24 | 31 | 7.963 | 95.5% | 0.846 |
| No25 | 574 | -0.021 | 49.0% | 0.871 |
| No26 | 42 | 1.312 | 82.7% | 1.005 |
| No27 | 83 | 0.985 | 80.9% | 0.956 |
| No28 | 88 | 1.147 | 83.0% | 0.974 |
| No29 | 243 | 1.949 | 86.8% | 1.146 |
| No30 | 32 | 1.228 | 85.9% | 1.527 |
| No31 | 99 | 0.264 | 59.3% | 1.303 |
| No32 | 22 | 6.623 | 80.0% | 1.061 |
| No33 | 36 | 7.731 | 93.8% | 0.911 |
| No34 | 61 | 7.269 | 91.1% | 0.949 |
| No35 | 99 | 1.819 | 93.2% | 0.960 |
| No36 | 13 | 7.498 | 95.5% | 1.781 |
| No37 | 68 | 1.082 | 73.1% | 1.439 |
| No38 | 64 | 0.919 | 78.7% | 0.807 |
| No39 | 178 | 1.968 | 92.5% | 0.923 |
| No40 | 108 | 1.442 | 74.9% | 0.980 |
| No41 | 81 | 1.791 | 93.3% | 1.351 |
| No42 | 33 | 0.494 | 63.9% | 1.325 |
| No43 | 60 | 0.680 | 72.7% | 4.466 |
| No44 | 33 | 0.485 | 65.5% | 0.878 |
| No45 | 100 | 1.203 | 85.0% | 1.307 |
| No46 | 110 | 1.789 | 89.8% | 1.099 |
| No47 | 116 | 7.042 | 82.1% | 1.042 |
| No48 | 144 | 1.191 | 80.0% | 0.888 |
| No49 | 96 | 1.754 | 82.8% | 0.755 |
| No50 | 39 | 1.381 | 88.3% | 1.252 |
| No51 | 76 | 7.287 | 91.7% | 0.955 |
| No52 | 107 | 0.620 | 71.7% | 0.850 |
| No53 | 98 | 1.375 | 86.1% | 0.802 |
| No54 | 20 | 0.738 | 74.2% | 0.908 |
| No55 | 113 | 1.249 | 80.4% | 0.802 |
| No56 | 37 | 6.907 | 80.0% | 0.883 |
| No57+58^*^ | 16 | 6.759 | 86.4% | 1.408 |
| No59 | 60 | 7.046 | 94.2% | 2.083 |
| No60 | 37 | 1.330 | 87.6% | 1.289 |
|  |  |  |  |  |
| **Total** | **5332** | **0.999** | **80.5%** | **0.984** |

AUC = area under the receiver-operating-characteristic curve; Exp./Obs. = Ratio of expected (or predicted) and observed outcomes.

Median AUC = 85.9 (IQR: 79.4% to 93.2%); Average calibration slope of 1.09 (95% CI: 0.87 to 1.31) based on a random-effects meta-analysis; Median ratio of expected/observed = 0.976 (IQR: 0.892 to 1.199).

***** For the logistic regression modelling procedure study No57 was combined with study No58 to one study since only two patients were included from study No58 for this cohort.

**Supplemental Table 10 Results of dataset-specific performance of the Combined COME-CCT-PTP calculator with CTA**

| **Study ID** | **Patients** | **Patients With CAD** | **Patients Without CAD** | **Study  Prevalence** | **AUC** | **Brier Score** | **Maximum**  **Brier Score** | **Scaled Brier Score** | **Discrimination Slope** |
| --- | --- | --- | --- | --- | --- | --- | --- | --- | --- |
| No1 | 49 | 30 | 19 | 61.2% | 100.0% | 0.049 | 0.237 | 0.793 | 0.667 |
| No2 | 99 | 35 | 64 | 35.4% | 93.9% | 0.090 | 0.229 | 0.606 | 0.496 |
| No3 | 150 | 59 | 91 | 39.3% | 95.1% | 0.085 | 0.239 | 0.644 | 0.541 |
| No4 | 80 | 39 | 41 | 48.8% | 95.8% | 0.089 | 0.250 | 0.643 | 0.516 |
| No5 | 24 | 9 | 15 | 37.5% | 98.5% | 0.059 | 0.234 | 0.749 | 0.582 |
| No6 | 67 | 29 | 38 | 43.3% | 91.4% | 0.115 | 0.245 | 0.530 | 0.455 |
| No7 | 170 | 84 | 86 | 49.4% | 94.4% | 0.109 | 0.250 | 0.564 | 0.458 |
| No8 | 127 | 46 | 81 | 36.2% | 94.6% | 0.103 | 0.231 | 0.554 | 0.417 |
| No9 | 210 | 180 | 30 | 85.7% | 72.6% | 0.157 | 0.122 | -0.282 | 0.218 |
| No10 | 65 | 22 | 43 | 33.8% | 65.8% | 0.264 | 0.224 | -0.181 | 0.077 |
| No11 | 129 | 67 | 62 | 51.9% | 86.1% | 0.139 | 0.250 | 0.445 | 0.408 |
| No12 | 29 | 11 | 18 | 37.9% | 93.9% | 0.103 | 0.235 | 0.564 | 0.436 |
| No13 | 75 | 50 | 25 | 66.7% | 91.2% | 0.111 | 0.222 | 0.499 | 0.492 |
| No14 | 26 | 19 | 7 | 73.1% | 98.5% | 0.069 | 0.197 | 0.647 | 0.657 |
| No15 | 265 | 136 | 129 | 51.3% | 89.4% | 0.123 | 0.250 | 0.510 | 0.460 |
| No16 | 99 | 31 | 68 | 31.3% | 88.6% | 0.127 | 0.215 | 0.410 | 0.463 |
| No17 | 50 | 20 | 30 | 40.0% | 59.4% | 0.298 | 0.240 | -0.242 | 0.045 |
| No18 | 230 | 72 | 158 | 31.3% | 68.2% | 0.255 | 0.215 | -0.187 | 0.194 |
| No19 | 8 | 3 | 5 | 37.5% | 100.0% | 0.040 | 0.234 | 0.828 | 0.638 |
| No20 | 41 | 24 | 17 | 58.5% | 92.2% | 0.118 | 0.243 | 0.514 | 0.465 |
| No21 | 75 | 17 | 58 | 22.7% | 87.8% | 0.114 | 0.175 | 0.352 | 0.431 |
| No22 | 15 | 5 | 10 | 33.3% | 100.0% | 0.033 | 0.222 | 0.853 | 0.648 |
| No23 | 32 | 15 | 17 | 46.9% | 97.3% | 0.083 | 0.249 | 0.668 | 0.555 |
| No24 | 31 | 20 | 11 | 64.5% | 94.3% | 0.067 | 0.229 | 0.706 | 0.537 |
| No25 | 574 | 274 | 300 | 47.7% | 61.7% | 0.293 | 0.249 | -0.176 | 0.092 |
| No26 | 42 | 22 | 20 | 52.4% | 87.7% | 0.139 | 0.249 | 0.445 | 0.385 |
| No27 | 83 | 39 | 44 | 47.0% | 87.1% | 0.142 | 0.249 | 0.430 | 0.438 |
| No28 | 88 | 44 | 44 | 50.0% | 86.8% | 0.139 | 0.250 | 0.443 | 0.391 |
| No29 | 243 | 101 | 142 | 41.6% | 90.9% | 0.114 | 0.243 | 0.532 | 0.449 |
| No30 | 32 | 6 | 26 | 18.8% | 94.2% | 0.079 | 0.152 | 0.478 | 0.465 |
| No31 | 99 | 38 | 61 | 38.4% | 77.1% | 0.214 | 0.237 | 0.095 | 0.277 |
| No32 | 22 | 12 | 10 | 54.5% | 85.0% | 0.152 | 0.248 | 0.388 | 0.398 |
| No33 | 36 | 20 | 16 | 55.6% | 95.2% | 0.081 | 0.247 | 0.670 | 0.563 |
| No34 | 61 | 33 | 28 | 54.1% | 88.3% | 0.119 | 0.248 | 0.523 | 0.449 |
| No35 | 99 | 43 | 56 | 43.4% | 97.3% | 0.071 | 0.246 | 0.712 | 0.575 |
| No36 | 13 | 2 | 11 | 15.4% | 95.5% | 0.068 | 0.130 | 0.475 | 0.687 |
| No37 | 68 | 26 | 42 | 38.2% | 83.8% | 0.171 | 0.236 | 0.276 | 0.304 |
| No38 | 64 | 43 | 21 | 67.2% | 80.8% | 0.167 | 0.220 | 0.241 | 0.324 |
| No39 | 178 | 93 | 85 | 52.2% | 96.5% | 0.075 | 0.249 | 0.701 | 0.579 |
| No40 | 108 | 69 | 39 | 63.9% | 81.1% | 0.148 | 0.231 | 0.359 | 0.322 |
| No41 | 81 | 18 | 63 | 22.2% | 98.3% | 0.055 | 0.173 | 0.681 | 0.668 |
| No42 | 33 | 14 | 19 | 42.4% | 72.6% | 0.228 | 0.244 | 0.066 | 0.187 |
| No43 | 60 | 5 | 55 | 8.3% | 76.4% | 0.162 | 0.076 | -1.123 | 0.242 |
| No44 | 33 | 21 | 12 | 63.6% | 76.6% | 0.197 | 0.231 | 0.147 | 0.261 |
| No45 | 100 | 29 | 71 | 29.0% | 91.7% | 0.108 | 0.206 | 0.476 | 0.481 |
| No46 | 110 | 44 | 66 | 40.0% | 95.4% | 0.095 | 0.240 | 0.603 | 0.563 |
| No47 | 116 | 63 | 53 | 54.3% | 84.9% | 0.133 | 0.248 | 0.465 | 0.386 |
| No48 | 144 | 95 | 49 | 66.0% | 83.2% | 0.134 | 0.224 | 0.405 | 0.404 |
| No49 | 96 | 90 | 6 | 93.8% | 82.8% | 0.094 | 0.059 | -0.605 | 0.407 |
| No50 | 39 | 11 | 28 | 28.2% | 91.6% | 0.108 | 0.202 | 0.466 | 0.404 |
| No51 | 76 | 40 | 36 | 52.6% | 95.2% | 0.082 | 0.249 | 0.669 | 0.542 |
| No52 | 107 | 59 | 48 | 55.1% | 83.5% | 0.176 | 0.247 | 0.290 | 0.347 |
| No53 | 98 | 45 | 53 | 45.9% | 91.4% | 0.106 | 0.248 | 0.572 | 0.511 |
| No54 | 20 | 11 | 9 | 55.0% | 78.8% | 0.189 | 0.248 | 0.237 | 0.320 |
| No55 | 113 | 90 | 23 | 79.6% | 83.0% | 0.154 | 0.162 | 0.049 | 0.345 |
| No56 | 37 | 27 | 10 | 73.0% | 95.0% | 0.123 | 0.197 | 0.377 | 0.385 |
| No57+58^*^ | 16 | 5 | 11 | 31.3% | 91.8% | 0.135 | 0.215 | 0.372 | 0.548 |
| No59 | 60 | 8 | 52 | 13.3% | 99.8% | 0.043 | 0.116 | 0.630 | 0.590 |
| No60 | 37 | 10 | 27 | 27.0% | 93.0% | 0.098 | 0.197 | 0.504 | 0.501 |
|  |  |  |  |  |  |  |  |  |  |
| **Total** | **5332** | **2573** | **2759** | **48.3%** | **86.0%** | **0.146** | **0.250** | **0.414** | **0.399** |

CAD = coronary artery disease; AUC = area under the receiver-operating-characteristic curve.

Median AUC=91.4% (IQR: 89.3% to 95.2%); Median Brier-Score = 0.114 (IQR: 0.084 to 0.150); Median Max Brier-Score = 0.235 (IQR: 0.215 to 0.248); Median Scaled Brier-Score= 0.476 (IQR: 0.321 to 0.618); Median Discrimination Slope = 0450 (IQR: 0.366 to 0.541).

* For the logistic regression modelling procedure study No57 was combined with study No58 to one study since only two patients were included from study No58 for this cohort.

**Supplemental Table 11 Internal-external cross-validation of dataset-specific performance of the Combined COME-CCT-PTP calculator with CTA**

| **Study ID** | **Patients** | **Calibration**  **Slope** | **AUC** | **Exp./Obs.** |
| --- | --- | --- | --- | --- |
| No1 | 49 | 32.241^+^ | 100.0% | 0.810 |
| No2 | 99 | 1.668 | 93.8% | 1.015 |
| No3 | 150 | 1.753 | 95.2% | 1.044 |
| No4 | 80 | 2.173 | 95.7% | 0.875 |
| No5 | 24 | 3.822 | 98.5% | 1.102 |
| No6 | 67 | 1.584 | 91.8% | 1.063 |
| No7 | 170 | 2.255 | 93.9% | 0.745 |
| No8 | 127 | 2.331 | 94.6% | 0.791 |
| No9 | 210 | 0.767 | 72.3% | 0.734 |
| No10 | 65 | 0.219 | 65.8% | 1.220 |
| No11 | 129 | 1.176 | 86.1% | 0.897 |
| No12 | 29 | 2.051 | 93.4% | 0.834 |
| No13 | 75 | 1.262 | 91.8% | 0.851 |
| No14 | 26 | 2.068 | 97.0% | 0.797 |
| No15 | 265 | 1.240 | 89.4% | 0.917 |
| No16 | 99 | 1.129 | 88.6% | 1.348 |
| No17 | 50 | 0.181 | 58.4% | 1.295 |
| No18 | 230 | 0.438 | 68.2% | 1.616 |
| No19 | 8 | 23.626^+^ | 100.0% | 1.159 |
| No20 | 41 | 1.343 | 92.2% | 1.009 |
| No21 | 75 | 1.173 | 88.1% | 1.379 |
| No22 | 15 | 26.011^+^ | 100.0% | 0.942 |
| No23 | 32 | 2.241 | 97.3% | 1.117 |
| No24 | 31 | 2.575 | 94.3% | 1.057 |
| No25 | 574 | 0.146 | 61.1% | 0.920 |
| No26^#^ | 42 | 1.492 | 87.7% | 0.816 |
| No27 | 83 | 0.985 | 87.1% | 1.010 |
| No28 | 88 | 1.180 | 86.4% | 0.942 |
| No29 | 243 | 1.814 | 90.8% | 1.045 |
| No30 | 32 | 1.500 | 94.2% | 1.330 |
| No31 | 99 | 0.624 | 76.8% | 1.266 |
| No32 | 22 | 0.975 | 85.0% | 0.960 |
| No33 | 36 | 1.839 | 94.8% | 0.856 |
| No34 | 61 | 1.341 | 88.3% | 0.972 |
| No35 | 99 | 2.153 | 97.3% | 1.046 |
| No36 | 13 | 2.078 | 95.5% | 1.828 |
| No37 | 68 | 1.395 | 83.7% | 1.236 |
| No38 | 64 | 0.811 | 80.0% | 0.857 |
| No39 | 178 | 1.894 | 96.6% | 0.942 |
| No40 | 108 | 1.186 | 80.6% | 1.002 |
| No41 | 81 | 1.901 | 98.3% | 1.401 |
| No42 | 33 | 0.546 | 71.1% | 1.177 |
| No43 | 60 | 0.640 | 75.6% | 3.572 |
| No44 | 33 | 0.630 | 76.6% | 1.057 |
| No45 | 100 | 1.300 | 91.8% | 1.220 |
| No46 | 110 | 1.688 | 95.4% | 1.210 |
| No47 | 116 | 1.713 | 85.4% | 0.932 |
| No48 | 144 | 1.051 | 83.2% | 0.909 |
| No49 | 96 | 1.814 | 84.0% | 0.743 |
| No50 | 39 | 1.600 | 91.2% | 1.138 |
| No51 | 76 | 2.140 | 95.2% | 0.921 |
| No52 | 107 | 0.819 | 83.2% | 0.970 |
| No53 | 98 | 1.309 | 91.4% | 0.937 |
| No54 | 20 | 0.756 | 78.8% | 1.116 |
| No55 | 113 | 1.323 | 83.4% | 0.675 |
| No56 | 37 | 4.275 | 94.4% | 0.743 |
| No57+58^*^ | 16 | 1.482 | 91.8% | 1.493 |
| No59 | 60 | 10.066^+^ | 99.8% | 1.509 |
| No60 | 37 | 1.189 | 93.3% | 1.097 |
|  |  |  |  |  |
| **Total** | **5332** | **1.061** | **86.0%** | **0.970** |

AUC = area under the receiver-operating-characteristic curve; Exp./Obs. = Ratio of expected (or predicted) and observed outcomes.

Median AUC = 91.5% (IQR: 83.6% to 95.0%); Average calibration slope of 1.28 (95% CI: 1.09 to 1.47) based on a random-effects meta-analysis; Median ratio of expected/observed 1.01(IQR: 0.903 to 1.194).

***** For the logistic regression modelling procedure study No57 was combined with study No58 to one study since only two patients were included from study No58 for this cohort.

^+^ The standard error of the estimates of the calibration slope for these studies was at least 1 400 times the estimates of this parameter except for study No59, where the standard error was almost half of the estimate.

^#^ The multivariable logistic regression model with dataset-specific random intercept failed to converge in R for these studies.

**Supplemental Table 12 Sensitivity analysis. Use of average intercepts according to Debray et al (2013).^2^ Internal-external cross-validation of study-specific performance of the Combined COME-CCT-PTP calculator and CTA**

| **Study ID** | **Patients** | **Calibration Slope** | **AUC** | **Exp./Obs.** |
| --- | --- | --- | --- | --- |
| No1 | 49 | 30,101^+^ | 100,0% | 0,815 |
| No2 | 99 | 1,553 | 93,8% | 1,007 |
| No3 | 150 | 1,632 | 95,2% | 1,041 |
| No4 | 80 | 2,023 | 95,7% | 0,876 |
| No5 | 24 | 3,534 | 98,5% | 1,093 |
| No6 | 67 | 1,473 | 91,8% | 1,062 |
| No7 | 170 | 2,106 | 93,9% | 0,743 |
| No8 | 127 | 2,171 | 94,6% | 0,778 |
| No9 | 210 | 0,726 | 72,3% | 0,743 |
| No10 | 65 | 0,203 | 65,8% | 1,197 |
| No11 | 129 | 1,098 | 86,1% | 0,901 |
| No12 | 29 | 1,907 | 93,4% | 0,823 |
| No13 | 75 | 1,178 | 91,8% | 0,859 |
| No14 | 26 | 1,948 | 97,0% | 0,805 |
| No15 | 265 | 1,158 | 89,4% | 0,920 |
| No16 | 99 | 1,055 | 88,6% | 1,336 |
| No17 | 50 | 0,167 | 58,4% | 1,302 |
| No18 | 230 | 0,412 | 68,2% | 1,618 |
| No19 | 8 | 21,865^+^ | 100,0% | 1,151 |
| No20 | 41 | 1,250 | 92,2% | 1,017 |
| No21 | 75 | 1,095 | 88,1% | 1,360 |
| No22 | 15 | 24,085^+^ | 100,0% | 0,933 |
| No23 | 32 | 2,083 | 97,3% | 1,120 |
| No24 | 31 | 2,414 | 94,3% | 1,065 |
| No25 | 574 | 0,133 | 61,1% | 0,918 |
| No26^#^ | 42 | 1,393 | 87,7% | 0,821 |
| No27 | 83 | 0,918 | 87,1% | 1,010 |
| No28 | 88 | 1,105 | 86,4% | 0,943 |
| No29 | 243 | 1,683 | 90,8% | 1,046 |
| No30 | 32 | 1,399 | 94,2% | 1,298 |
| No31 | 99 | 0,583 | 76,8% | 1,267 |
| No32 | 22 | 0,911 | 85,0% | 0,969 |
| No33 | 36 | 1,713 | 94,8% | 0,861 |
| No34 | 61 | 1,248 | 88,3% | 0,976 |
| No35 | 99 | 1,999 | 97,3% | 1,044 |
| No36 | 13 | 1,958 | 95,5% | 1,786 |
| No37 | 68 | 1,296 | 83,7% | 1,243 |
| No38 | 64 | 0,758 | 80,0% | 0,863 |
| No39 | 178 | 1,761 | 96,6% | 0,946 |
| No40 | 108 | 1,106 | 80,6% | 1,014 |
| No41 | 81 | 1,775 | 98,3% | 1,378 |
| No42 | 33 | 0,512 | 71,1% | 1,185 |
| No43 | 60 | 0,603 | 75,6% | 3,534 |
| No44 | 33 | 0,586 | 76,6% | 1,063 |
| No45 | 100 | 1,213 | 91,8% | 1,211 |
| No46 | 110 | 1,577 | 95,4% | 1,210 |
| No47 | 116 | 1,592 | 85,4% | 0,940 |
| No48 | 144 | 0,982 | 83,2% | 0,919 |
| No49 | 96 | 1,706 | 84,0% | 0,753 |
| No50 | 39 | 1,491 | 91,2% | 1,123 |
| No51 | 76 | 1,989 | 95,2% | 0,926 |
| No52 | 107 | 0,763 | 83,2% | 0,972 |
| No53 | 98 | 1,223 | 91,4% | 0,931 |
| No54 | 20 | 0,704 | 78,8% | 1,121 |
| No55 | 113 | 1,247 | 83,4% | 0,681 |
| No56 | 37 | 3,948 | 94,4% | 0,749 |
| No57+58^*^ | 16 | 1,394 | 91,8% | 1,496 |
| No59 | 60 | 9,284^+^ | 99,8% | 1,467 |
| No60 | 37 | 1,112 | 93,3% | 1,088 |
|  |  |  |  |  |
| **Total** | **5332** | **0,991** | **86,0%** | **0,972** |

AUC = area under the receiver-operating-characteristic curve. Exp./Obs. = Ratio of expected (or predicted) and observed outcomes

Median AUC = 91.5% (IQR: 83.6 to 95.0%); Average calibration slope of 1.19 (95% CI: 1.02 to 1.37) based on random-effects meta-analysis; Median ratio of expected/observed 1.014 (IQR: 0.909 to 1.191).

***** For the logistic regression modelling procedure study No57 was combined with study No58 to one study since only two patients were included from study No58 for this cohort.

^+^ The standard error of the estimates of the calibration slope for these studies was at least 1 400 times the estimates of this parameter. except for study No59, where the standard error was almost half of the estimate.

^#^ The multivariable logistic regression model with dataset-specific random intercept failed to converge in R for these studies.

**Supplemental Table 13 Results of pragmatic clinical prediction stratified by symptoms**

|  | **All patients** | **Typical angina** | **Atypical angina** | **Nonanginal chest discomfort** | **Other**  **chest discomfort** |
| --- | --- | --- | --- | --- | --- |
| AUC | 67.9% | 65.9% | 67.3% | 61.5% | 64.4% |
| Brier score* | 0.226 | 0.221 | 0.221 | 0.233 | 0.237 |
| Discrimination slope† | 0.107 | 0.063 | 0.069 | 0.046 | 0.051 |
| Threshold probability‡ | 0.449 | 0.635 | 0.399 | 0.285 | 0.347 |
| Accuracy§ | 62.5% | 64.6% | 62.4% | 54.6% | 61.1% |
| Misclassification\|\| | 37.5% | 35.4% | 37.6% | 45.4% | 38.9% |
| Sensitivity¶ | 65.5% | 66.7% | 66.1% | 85.8% | 63.6% |
| Specificity** | 59.7% | 61.3% | 59.8% | 33.3% | 59.3% |

AUC=area under the receiver-operating-characteristic curve.

*Brier score measuring the accuracy of probabilistic predictions, i.e., the average of the squared differences between actual outcome and predicted probabilities (prediction error)

† Discrimination slope=Difference in mean of predicted probabilities between outcomes

‡Optimal threshold/cut-off for predicted probabilities based on Youden’s index

§Accuracy, i.e., the fraction of correct classification based on the optimal threshold/cut-off for predicted probabilities

|| Misclassification = 100% - accuracy.

¶Sensitivity based on the optimal threshold/cut-off for predicted probabilities

**Specificity based on the optimal threshold/cut-off for predicted probabilities

**Supplemental Table 14 Results of prediction model including CTA alone stratified by symptoms**

|  | **All patients** | **Typical angina** | **Atypical angina** | **Nonanginal chest discomfort** | **Other chest discomfort** |
| --- | --- | --- | --- | --- | --- |
| AUC | 80.5% | 76.9% | 81.5% | 82.1% | 86.8% |
| Brier score* | 0.156 | 0.180 | 0.151 | 0.147 | 0.120 |
| Discrimination slope† | 0.359 | 0.315 | 0.370 | 0.377 | 0.432 |
| Threshold probability‡ | 0.439 | 0.439 | 0.439 | 0.439 | 0.439 |
| Accuracy§ | 80.3% | 78.1% | 79.8% | 80.5% | 85.5% |
| Misclassification\|\| | 19.7% | 21.9% | 20.2% | 19.5% | 14.5% |
| Sensitivity¶ | 87.5% | 82.5% | 90.3% | 90.7% | 95.1% |
| Specificity** | 73.6% | 71.2% | 72.7% | 73.5% | 78.5% |

AUC=area under the receiver-operating-characteristic curve.

* Brier score measuring the accuracy of probabilistic predictions, i.e. it is the average of the squared differences between actual outcome and predicted probabilities (prediction error).

†Discrimination slope=Difference in mean of predicted probabilities between outcomes.

‡Optimal threshold/cut-off for predicted probabilities based on Youden’s index.

§Accuracy, i.e., the fraction of right classification based on the optimal threshold/cut-off for predicted probabilities

||Misclassification = 100% - accuracy.

¶Sensitivity based on the optimal threshold/cut-off for predicted probabilities.

**Specificity based on the optimal threshold/cut-off for predicted probabilities.

**Supplemental Table 15 Results of COME-CCT-PTP calculator with CTA stratified by symptoms**

|  | **All patients** | **Typical angina** | **Atypical angina** | **Nonanginal chest discomfort** | **Other**  **chest discomfort** |
| --- | --- | --- | --- | --- | --- |
| AUC | 86.0% | 81.5% | 86.6% | 85.8% | 89.1% |
| Brier score* | 0.146 | 0.163 | 0.141 | 0.141 | 0.126 |
| Discrimination slope† | 0.400 | 0.344 | 0.379 | 0.373 | 0.404 |
| Threshold probability‡ | 0.379 | 0.651 | 0.386 | 0.458 | 0.245 |
| Accuracy§ | 80.5% | 78.2% | 80.1% | 81.7% | 85.5% |
| Misclassification\|\| | 19.5% | 21.8% | 19.9% | 18.3% | 14.5% |
| Sensitivity¶ | 88.9% | 82.4% | 90.2% | 88.6% | 95.1% |
| Specificity** | 72.6% | 71.6% | 73.3% | 76.9% | 78.5% |

AUC=area under the receiver-operating-characteristic curve.

* Brier score measuring the accuracy of probabilistic predictions, i.e. it is the average of the squared differences between actual outcome and predicted probabilities (prediction error).

†Discrimination slope=Difference in mean of predicted probabilities between outcomes.

‡Optimal threshold/cut-off for predicted probabilities based on Youden’s index.

§Accuracy, i.e., the fraction of right classification based on the optimal threshold/cut-off for predicted probabilities

||Misclassification = 100% - accuracy.

¶Sensitivity based on the optimal threshold/cut-off for predicted probabilities.

**Specificity based on the optimal threshold/cut-off for predicted probabilities.

**Supplemental Table 16 Sensitivity analysis. Use of average intercepts according to Debray et al (2013).^2^ Results of pragmatic clinical prediction stratified by symptoms.**

|  | **All patients** | **Typical angina** | **Atypical angina** | **Nonanginal chest discomfort** | **Other**  **chest discomfort** |
| --- | --- | --- | --- | --- | --- |
| AUC | 67.9% | 65.9% | 67.3% | 61.5% | 64.4% |
| Brier score* | 0.227 | 0.222 | 0.220 | 0.235 | 0.240 |
| Discrimination slope† | 0.118 | 0.070 | 0.077 | 0.050 | 0.055 |
| Threshold probability‡ | 0.443 | 0.653 | 0.385 | 0.260 | 0.328 |
| Accuracy§ | 62.5% | 64.6% | 62.4% | 54.6% | 61.1% |
| Misclassification\|\| | 37.5% | 35.4% | 37.6% | 45.4% | 38.9% |
| Sensitivity¶ | 65.5% | 66.7% | 66.1% | 85.8% | 63.6% |
| Specificity** | 59.7% | 61.3% | 59.8% | 33.3% | 59.3% |

AUC=area under the receiver-operating-characteristic curve.

* Brier score measuring the accuracy of probabilistic predictions, i.e. it is the average of the squared differences between actual outcome and predicted probabilities (prediction error).

†Discrimination slope=Difference in mean of predicted probabilities between outcomes.

‡Optimal threshold/cut-off for predicted probabilities based on Youden’s index.

§Accuracy, i.e., the fraction of right classification based on the optimal threshold/cut-off for predicted probabilities

||Misclassification = 100% - accuracy.

¶Sensitivity based on the optimal threshold/cut-off for predicted probabilities.

**Specificity based on the optimal threshold/cut-off for predicted probabilities.

**Supplemental Table 17 Sensitivity analysis. Use of average intercepts according to Debray et al (2013).^2^ Results of the prediction model including CTA alone**

|  | **All patients** | **Typical angina** | **Atypical angina** | **Nonanginal chest discomfort** | **Other**  **chest discomfort** |
| --- | --- | --- | --- | --- | --- |
| AUC | 80.5% | 76.9% | 81.5% | 82.1% | 86.8% |
| Brier score* | 0.155 | 0.180 | 0.151 | 0.147 | 0.119 |
| Discrimination slope† | 0.376 | 0.331 | 0.388 | 0.396 | 0.453 |
| Threshold probability‡ | 0.439 | 0.439 | 0.439 | 0.439 | 0.439 |
| Accuracy§ | 80.3% | 78.1% | 79.8% | 80.5% | 85.5% |
| Misclassification\|\| | 19.7% | 21.9% | 20.2% | 19.5% | 14.5% |
| Sensitivity¶ | 87.5% | 82.5% | 90.3% | 90.7% | 95.1% |
| Specificity** | 73.6% | 71.2% | 72.7% | 73.5% | 78.5% |

AUC=area under the receiver-operating-characteristic curve.

* Brier score measuring the accuracy of probabilistic predictions, i.e. it is the average of the squared differences between actual outcome and predicted probabilities (prediction error).

†Discrimination slope=Difference in mean of predicted probabilities between outcomes.

‡Optimal threshold/cut-off for predicted probabilities based on Youden’s index.

§Accuracy, i.e., the fraction of right classification based on the optimal threshold/cut-off for predicted probabilities

||Misclassification = 100% - accuracy.

¶Sensitivity based on the optimal threshold/cut-off for predicted probabilities.

**Specificity based on the optimal threshold/cut-off for predicted probabilities.

**Supplemental Table 18 Sensitivity analysis. Use of average intercepts according to Debray et al (2013).^2^ Results of pragmatic clinical prediction including CTA stratified by symptoms**

|  | **All patients** | **Typical angina** | **Atypical angina** | **Nonanginal chest discomfort** | **Other**  **chest discomfort** |
| --- | --- | --- | --- | --- | --- |
| AUC | 86.0% | 81.5% | 86.6% | 85.8% | 89.1% |
| Brier score* | 0.146 | 0.164 | 0.140 | 0.140 | 0.125 |
| Discrimination slope† | 0.415 | 0.360 | 0.394 | 0.388 | 0.419 |
| Threshold probability‡ | 0.369 | 0.663 | 0.376 | 0.454 | 0.229 |
| Accuracy§ | 80.5% | 78.2% | 80.1% | 81.7% | 85.5% |
| Misclassification\|\| | 19.5% | 21.8% | 19.9% | 18.3% | 14.5% |
| Sensitivity¶ | 88.9% | 82.4% | 90.2% | 88.6% | 95.1% |
| Specificity** | 72.6% | 71.6% | 73.3% | 76.9% | 78.5% |

AUC=area under the receiver-operating-characteristic curve.

* Brier score measuring the accuracy of probabilistic predictions, i.e. it is the average of the squared differences between actual outcome and predicted probabilities (prediction error).

†Discrimination slope=Difference in mean of predicted probabilities between outcomes.

‡Optimal threshold/cut-off for predicted probabilities based on Youden’s index.

§Accuracy, i.e., the fraction of right classification based on the optimal threshold/cut-off for predicted probabilities

||Misclassification = 100% - accuracy.

¶Sensitivity based on the optimal threshold/cut-off for predicted probabilities.

**Specificity based on the optimal threshold/cut-off for predicted probabilities.


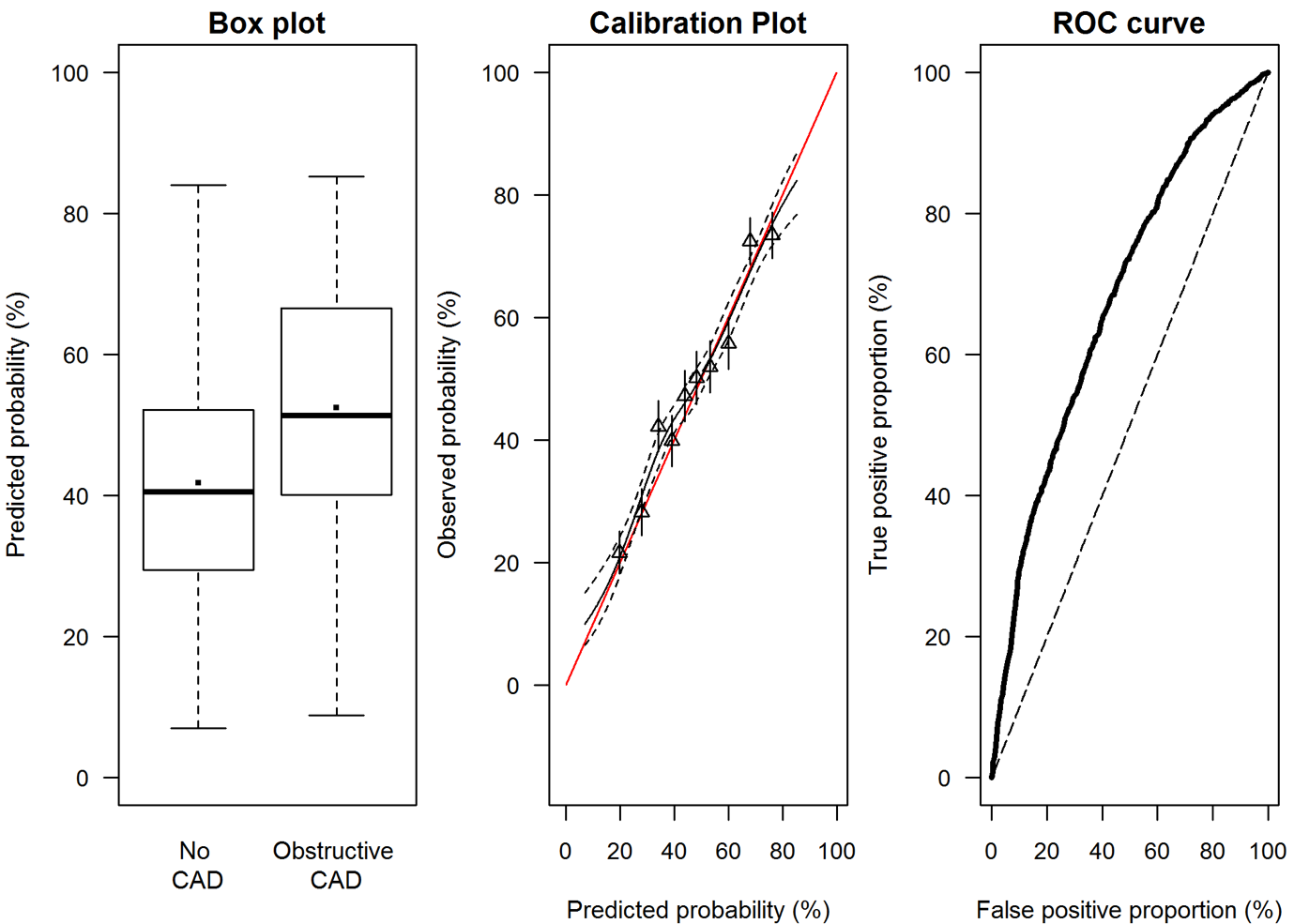

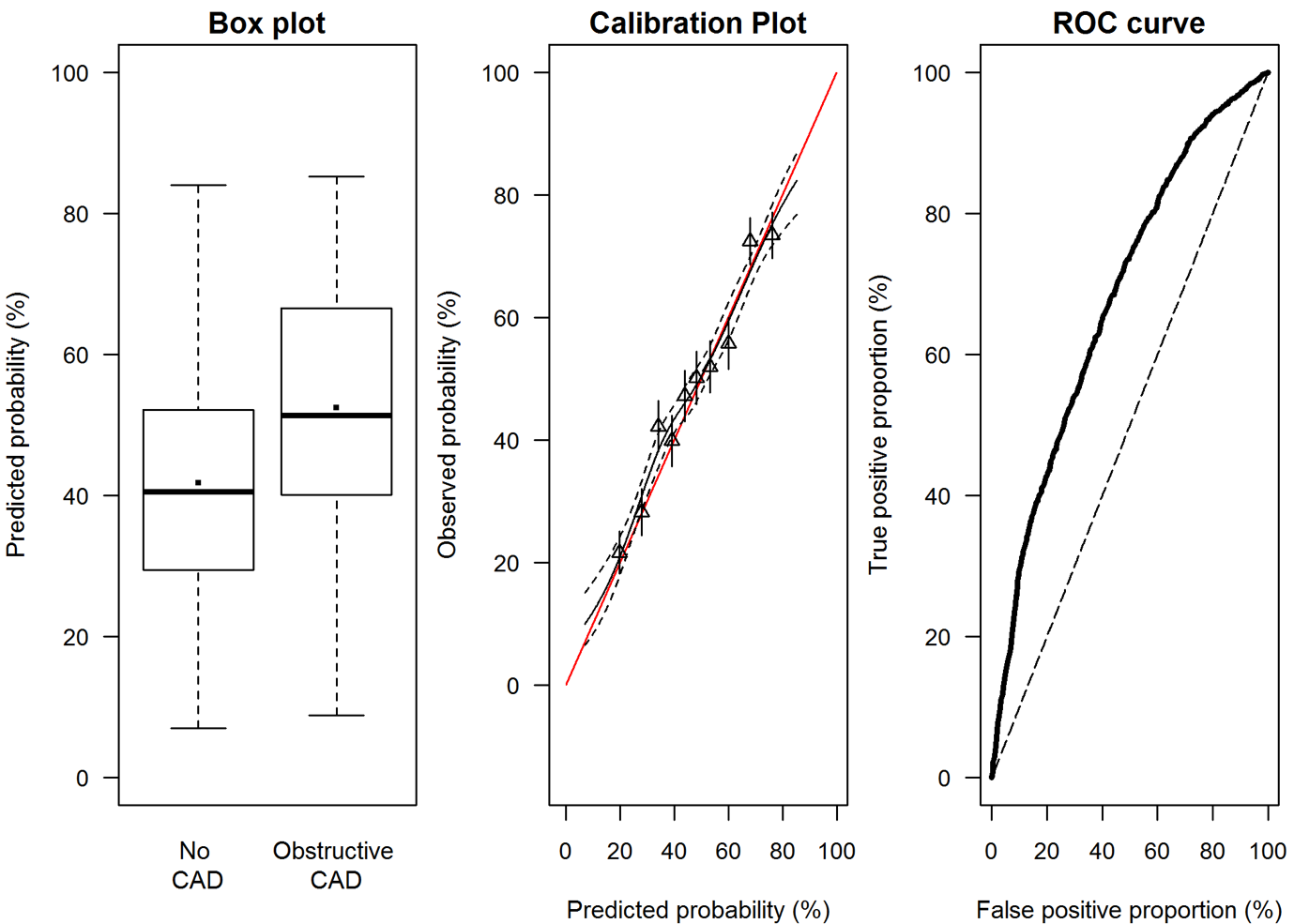


**Supplemental Figure 1 Discrimination and calibration plots for the COME-CCT-PTP calculator**

Boxplot of predicted probabilities (left) and ROC curve (right). The boxplot representation indicates the discriminatory ability of the prediction model. The squares in the boxplots represent mean predicted probabilities for presence versus absence of obstructive CAD on ICA. The difference of these mean values corresponds to the discrimination slope. The calibration plot is a graphical illustration of the Hosmer-Lemeshow goodness-of-fit test plotting observed probabilities vs. predicted probabilities grouped by decile of predicted probabilities.

**
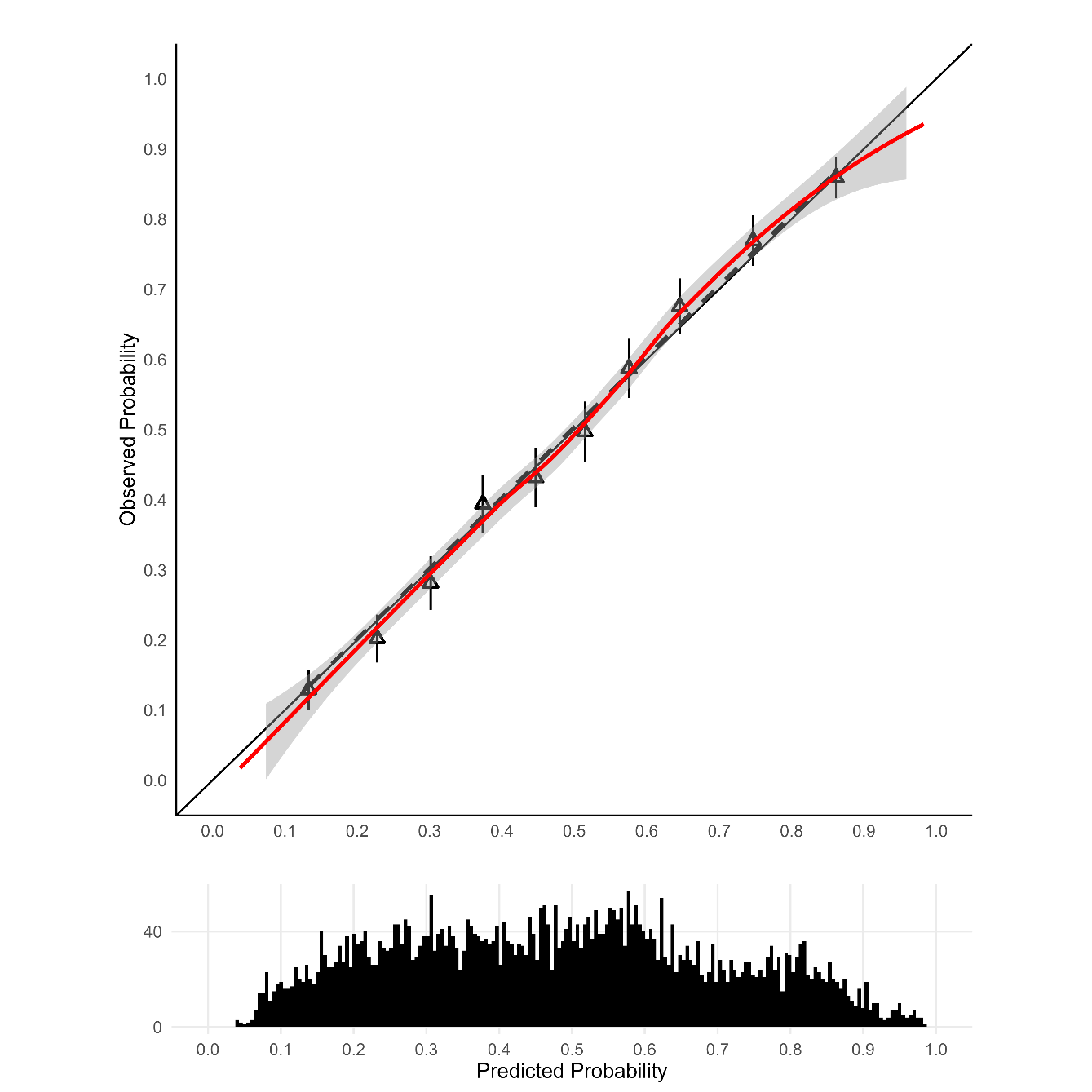
**

**Supplemental Figure 2 Calibration plots for the COME-CCT-PTP calculator**

The calibration plot is a graphical illustration of the Hosmer-Lemeshow goodness-of-fit test plotting observed probabilities vs. predicted probabilities grouped by decile of predicted probabilities. A better discriminating prediction model has more spread between deciles (compare Appendix Figure 7) than a poorly discriminating prediction model.


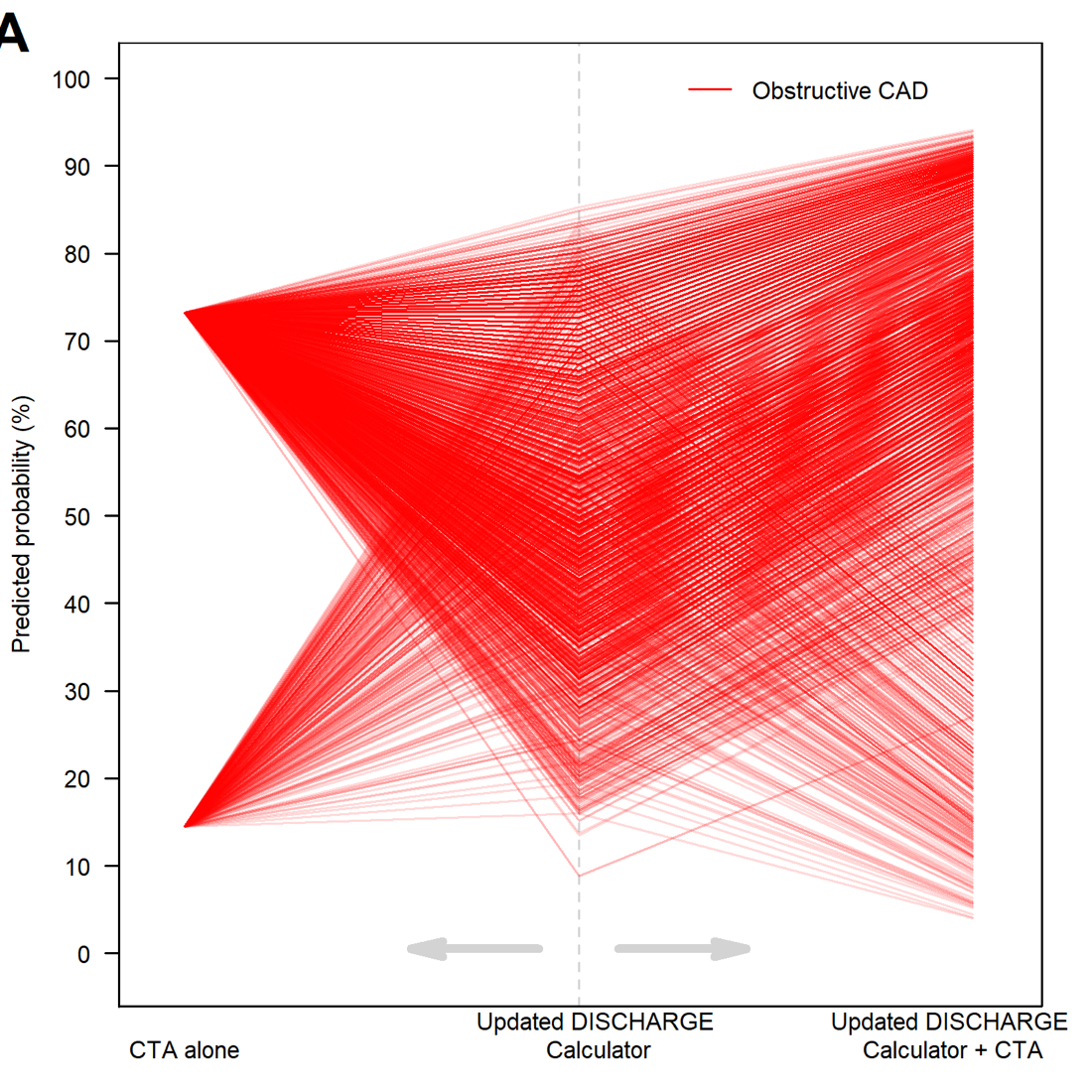


**CTA alone**

**COME CCT PTP**

**COME CCT PTP + CTA
+ CCTA**


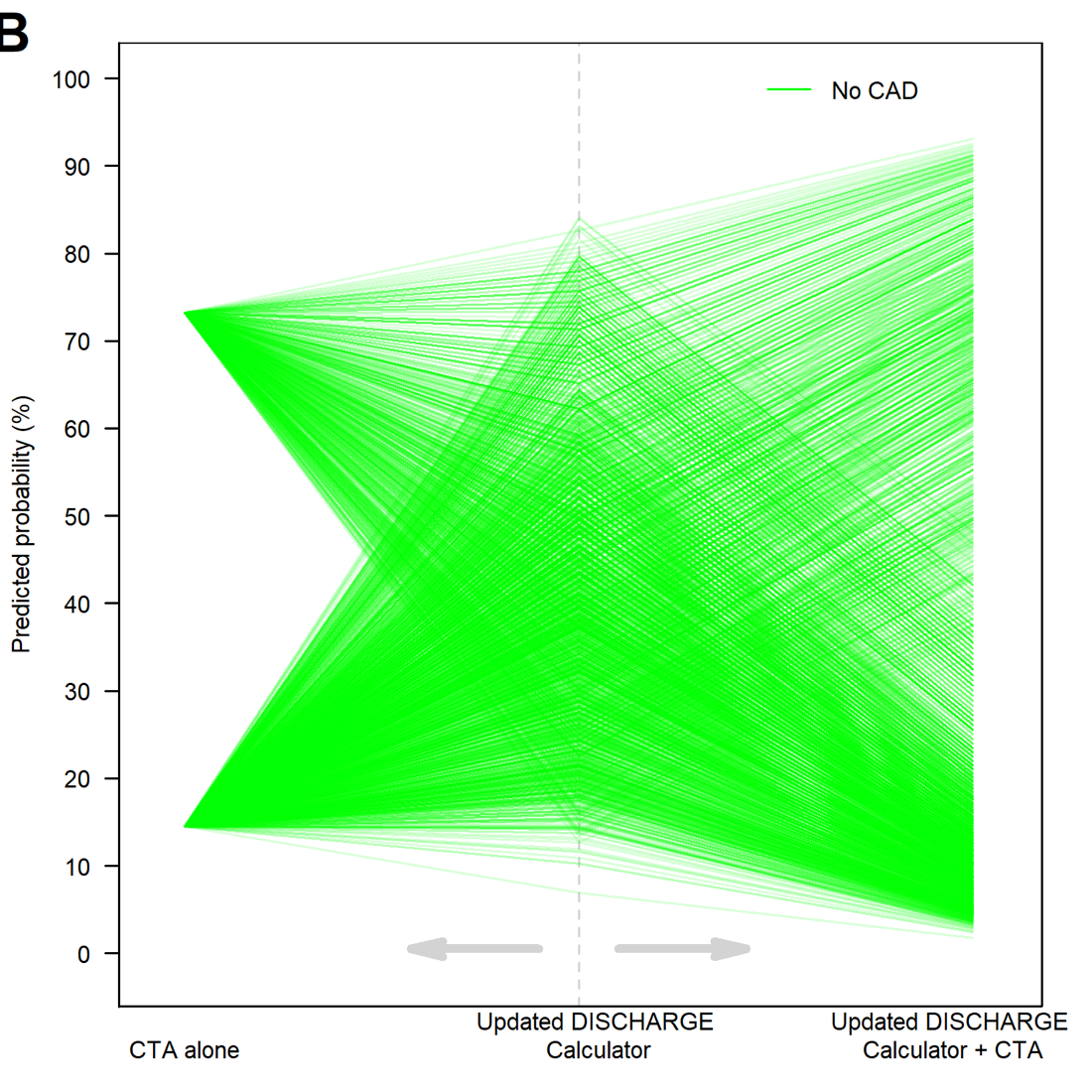


**CTA alone**

**COME CCT PTP**

**COME CCT PTP + CTA
+ CCTA**

**Supplemental Figure 3 Discrimination of patients by COME-CCT Pretest Probability Calculator, CTA alone, and combination of COME-CCT Pretest Probability Calculator and CTA**

Panel A shows the discrimination by the **COME-CCT-PTP calculator**, CTA alone, and combination of **COME-CCT-PTP calculator** and CTA in patients with obstructive CAD and Panel B shows the discrimination results in patients without obstructive CAD.


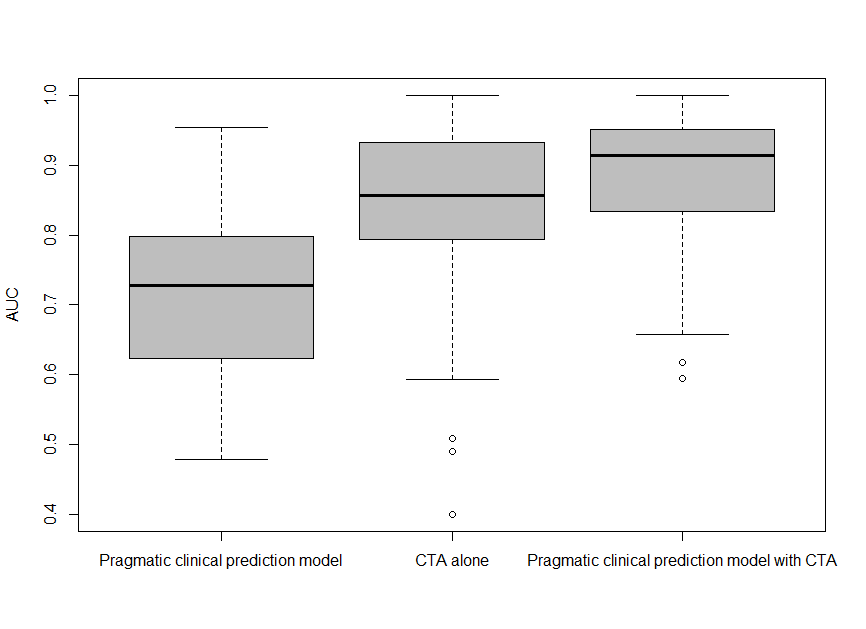


**Supplemental Figure 4 Boxplots of AUC for pragmatic clinical prediction models in individual studies**

AUC shown for the individual studies, which were fairly heterogeneous for pragmatic clinical prediction models with a median AUC of 72.3% (IQR: 61.9 to 79.9%). Heterogeneity across studies was relevantly reduced for the clinical prediction models with CTA (median AUC of 91.5% (IQR: 83.6 to 95.0%). The individual AUCs are calculated based on the respective dataset-specific results for pragmatic clinical prediction models with and without CTA (Appendix Tables 4, 7 and 10).


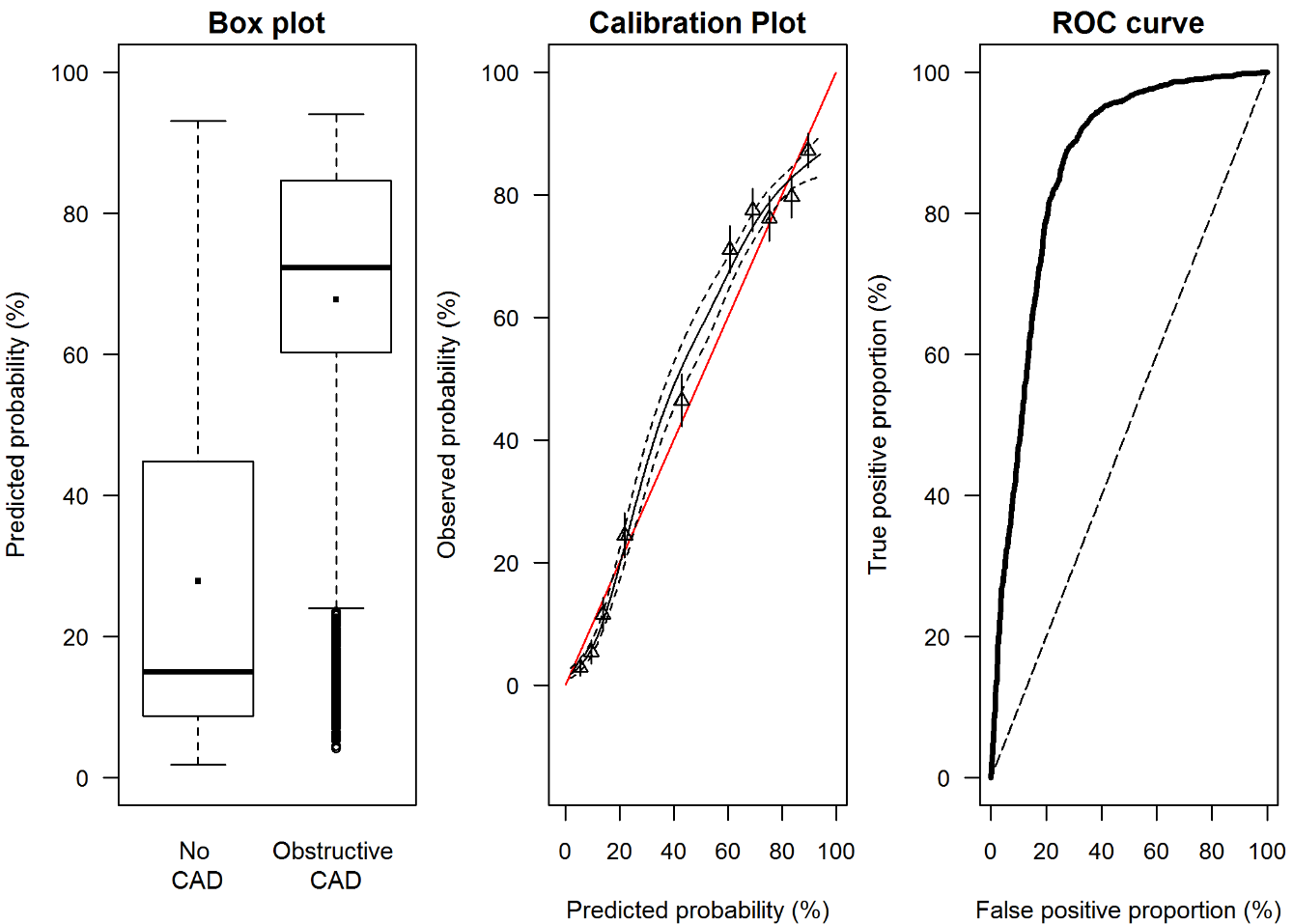

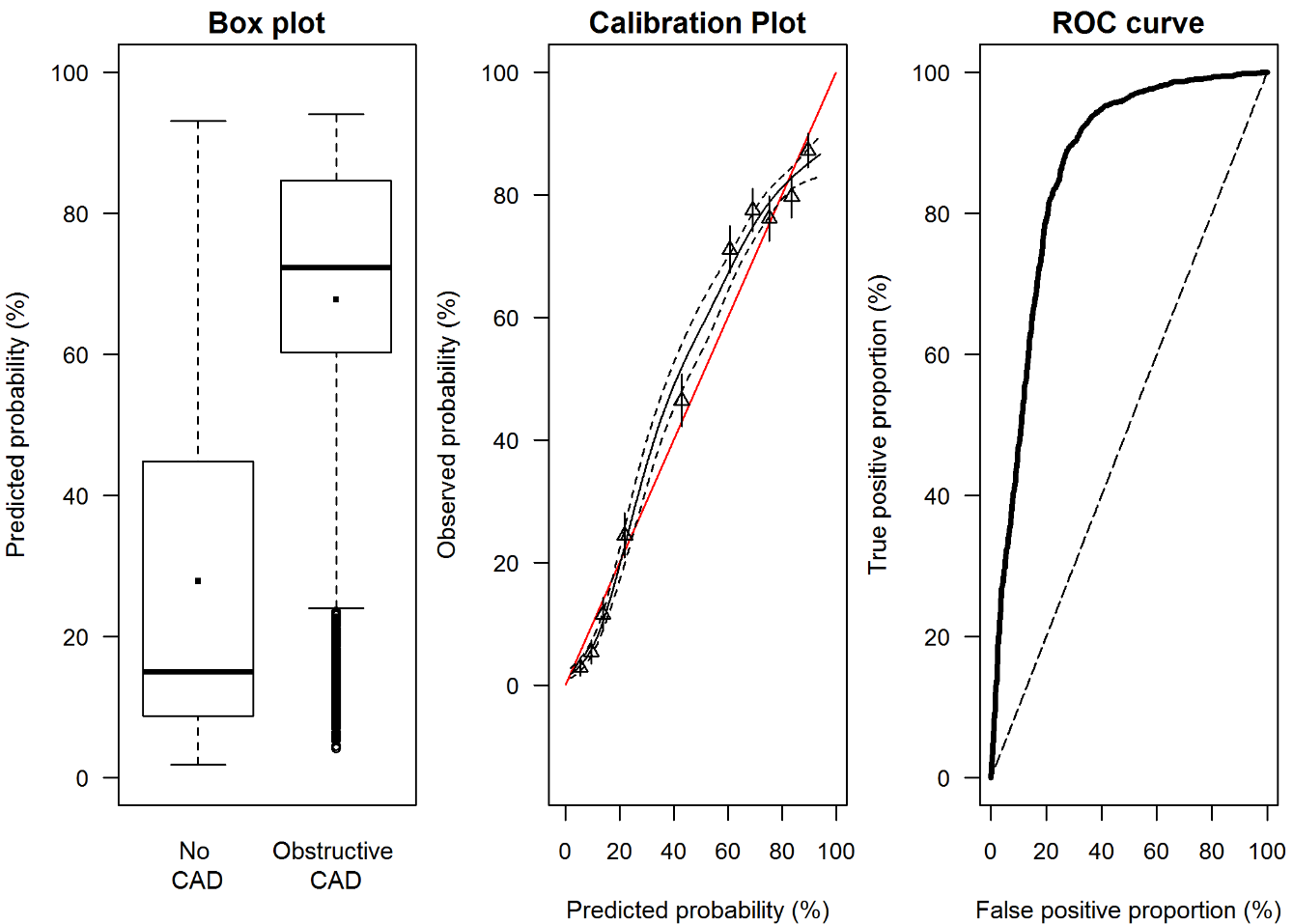


**Supplemental Figure 5 Discrimination and calibration plots for the combined COME-CCT-PTP calculator with CTA**

Boxplot of predicted probabilities (left), calibration plot (middle) and ROC curve (right).

The boxplot representation indicates the discriminatory ability of the prediction model. The squares in the boxplots represent mean predicted probabilities for presence versus absence of obstructive CAD. The difference of these mean values corresponds to the discrimination slope. The calibration plot is a graphical illustration of the Hosmer-Lemeshow goodness-of-fit test plotting observed probabilities vs. predicted probabilities grouped by decile of predicted probabilities. A better discriminating prediction model has more spread between deciles than a poorly discriminating prediction model (see Appendix Figure 8).


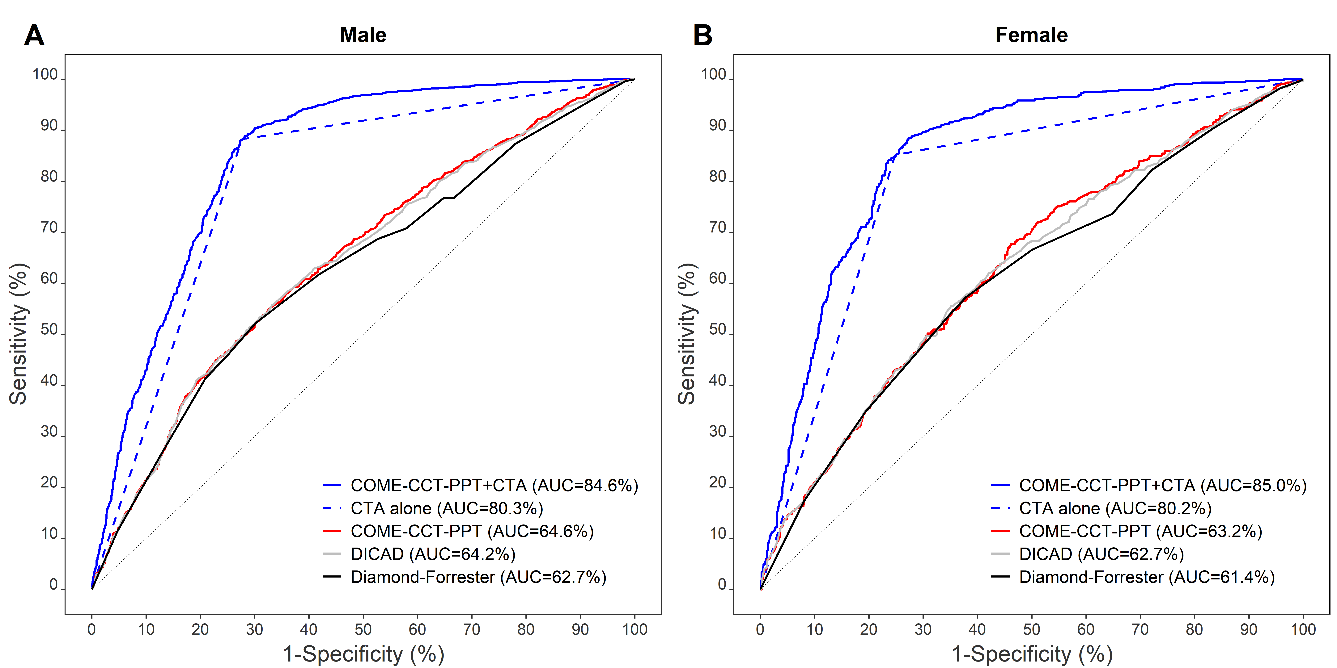


**Supplemental Figure 6 Discriminative Ability of Prediction Models Depending on Sex**

Results for the prediction model including CTA alone (dashed blue), for the original (black) and updated Diamond-Forrester results (grey) are shown for comparison. Panel A shows the ROC curves describing the clinical probability prediction models for male patients. The **COME-CCT-PTP calculator** with CTA (blue, 0.85, 0.84 to 0.86) improved discrimination compared with the **CTA alone model** (blue dashed line, 0.80, 0.79 to 0.81) and **COME-CCT-PTP calculator** alone (red, 0.65, 0.63 to 0.66). Panel B shows that the models have consistent and similar discrimination performance in female patients.

**
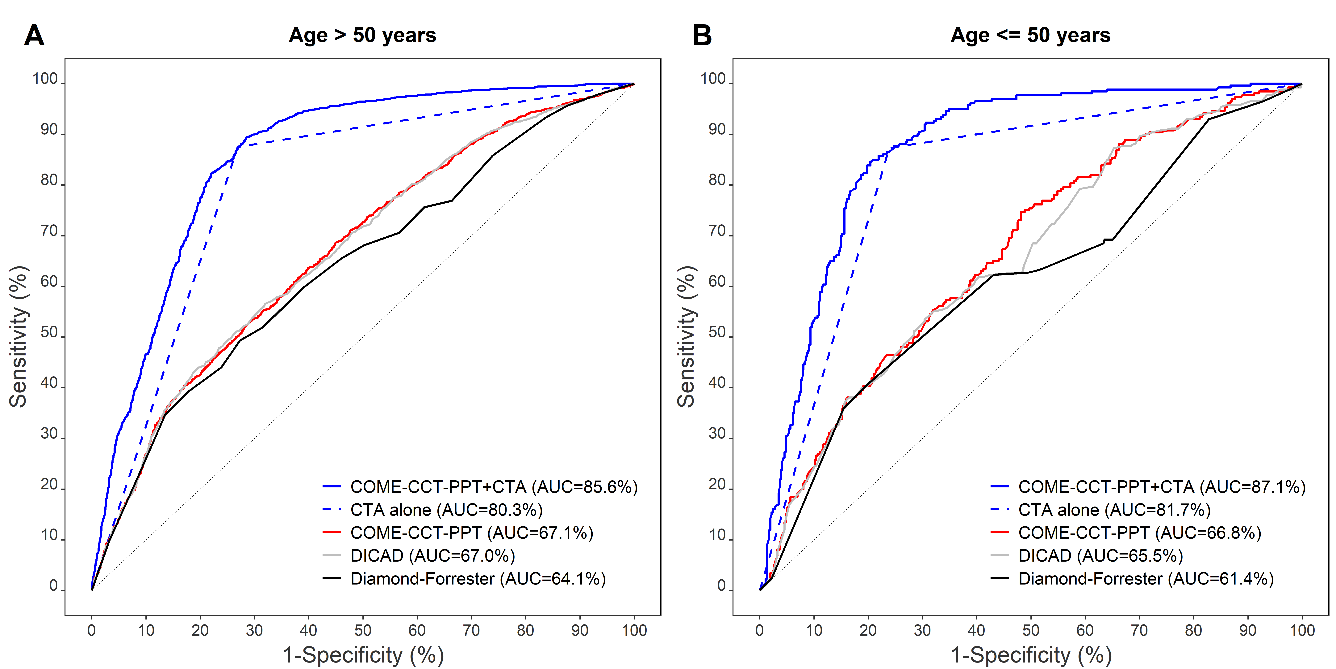
**

**Supplemental Figure 7 Discriminative Ability of Prediction Models Depending on Age**

Results for the prediction model including CTA alone (dashed blue), for the original (black) and updated Diamond-Forrester results (grey) are shown for comparison. Panel A shows the ROC curves describing the clinical probability prediction models for patients more than 50 years old. The **COME-CCT-PTP calculator** including CTA (blue, 0.86, 0.84 to 0.87) improved discrimination compared with the **CTA alone model** (blue dashed line, 0.80, 0.79 to 0.81) and with the **COME-CCT-PTP calculator** alone (red, 0.67, 0.66 to 0.68). Panel B shows that the models performed slightly better in patients less than 50 years old.

**
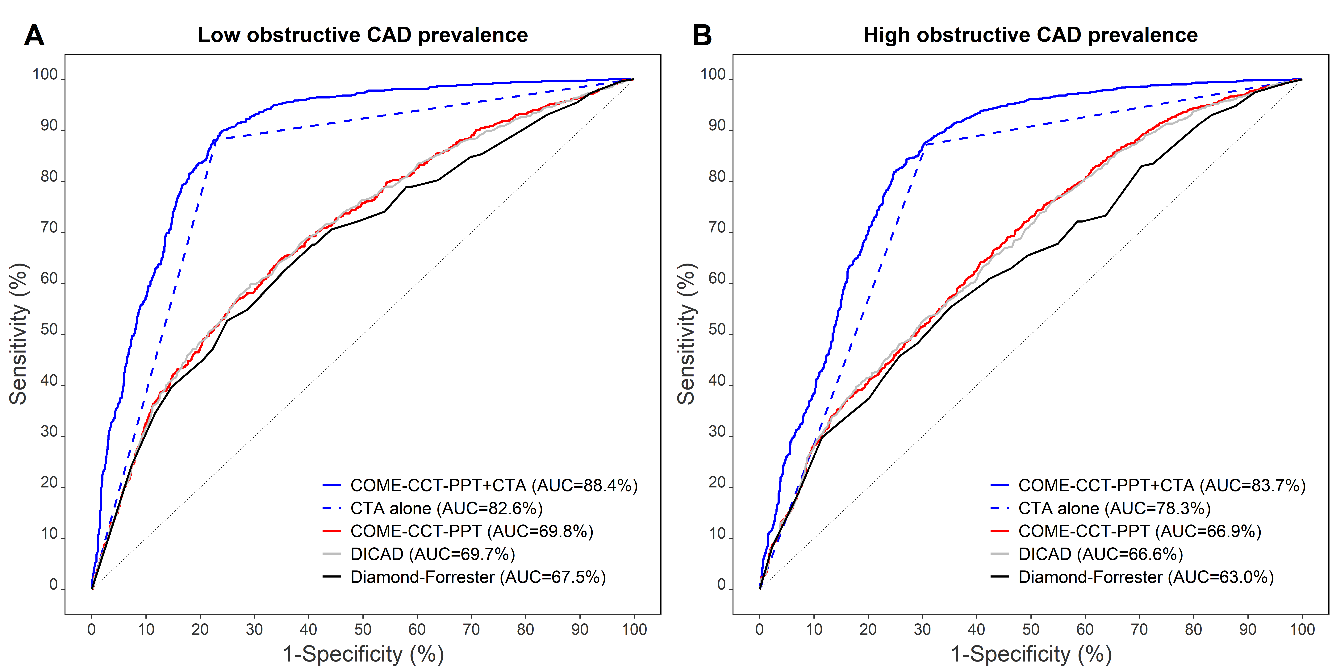
**

**Supplemental Figure 8 Discriminative Ability of Prediction Models Depending on Obstructive CAD Prevalence**

Results for the prediction model including CTA alone (dashed blue), for the original (black) and updated Diamond-Forrester results (grey) are shown for comparison. Panel A shows the ROC curves describing the clinical probability prediction models for patients drawn from studies with low CAD prevalence (<= 45%), while panel B describing the clinical probability prediction models for patients drawn from studies with high CAD prevalence (> 45%). The prevalence cut-off was based on the median CAD prevalence of included studies. The **COME-CCT-PTP calculator** showed better discriminative ability in case of lower CAD prevalence compared to the higher CAD prevalence (red lines, 0.70 vs 0.67). likewise the CTA alone model performed better in the case of lower CAD prevalence (blue dashed lines, 0.83 vs. 0.78), which resulted in better discriminative ability of **COME-CCT-PTP calculator** in case low CAD prevalence (blue line, 0.88, 0.87 to 0.86) compared with higher CAD prevalence (blue line, 0.84, 0.83 to 0.85).

**SUPPLEMENTAL REFERENCES**

1. Genders TS, Steyerberg EW, Alkadhi H, Leschka S, Desbiolles L, Nieman K, et al. A clinical prediction rule for the diagnosis of coronary artery disease: validation, updating, and extension. European heart journal. 2011.

2. Debray TP, Moons KG, Ahmed I, Koffijberg H, Riley RD. A framework for developing, implementing, and evaluating clinical prediction models in an individual participant data meta-analysis. Stat Med. 2013;32(18):3158-80.

**INCLUDED STUDIES***

1. Alkadhi H, Scheffel H, Desbiolles L, et al. Dual-source computed tomography coronary angiography: influence of obesity, calcium load, and heart rate on diagnostic accuracy. *European heart journal* 2008; **29**(6): 766-76.

2. Alkadhi H, Stolzmann P, Desbiolles L, et al. Low-dose, 128-slice, dual-source CT coronary angiography: accuracy and radiation dose of the high-pitch and the step-and-shoot mode. *Heart* 2010; **96**(12): 933-8.

3. Andreini D, Pontone G, Bartorelli AL, et al. Comparison of the diagnostic performance of 64-slice computed tomography coronary angiography in diabetic and non-diabetic patients with suspected coronary artery disease. *Cardiovascular diabetology* 2010; **9**: 80.

4. Andreini D, Pontone G, Bartorelli AL, et al. Sixty-four-slice multidetector computed tomography: an accurate imaging modality for the evaluation of coronary arteries in dilated cardiomyopathy of unknown etiology. *Circulation Cardiovascular imaging* 2009; **2**(3): 199-205.

5. Andreini D, Pontone G, Pepi M, et al. Diagnostic accuracy of multidetector computed tomography coronary angiography in patients with dilated cardiomyopathy. *Journal of the American College of Cardiology* 2007; **49**(20): 2044-50.

6. Arnoldi E, Ramos-Duran I, Abro JA, et al. [Coronary CT angiography using prospective ECG triggering: high diagnostic accuracy with low radiation dose]. *Der Radiologe* 2010; **50**(6): 500-6.

7. Bettencourt N, Rocha J, Carvalho M, et al. Multislice computed tomography in the exclusion of coronary artery disease in patients with presurgical valve disease. *Circulation Cardiovascular imaging* 2009; **2**(4): 306-13.

8. Bonmassari R, Muraglia S, Centonze M, Coser D, Stoppa G, Disertori M. Noninvasive detection of coronary artery stenosis with 16-slice spiral computed tomography in a population at low to moderate risk for coronary artery disease. *Journal of cardiovascular medicine* 2006; **7**(11): 817-25.

9. Chen CC, Chen CC, Hsieh IC, et al. The effect of calcium score on the diagnostic accuracy of coronary computed tomography angiography. *The international journal of cardiovascular imaging* 2011; **27 Suppl 1**: 37-42.

10. Chow BJ, Dennie C, Hoffmann U, et al. Comparison of computed tomographic angiography versus rubidium-82 positron emission tomography for the detection of patients with anatomical coronary artery disease. *The Canadian journal of cardiology* 2007; **23**(10): 801-7.

11. Dewey M, Teige F, Schnapauff D, et al. Noninvasive detection of coronary artery stenoses with multislice computed tomography or magnetic resonance imaging. *Annals of internal medicine* 2006; **145**(6): 407-15.

12. Dewey M, Zimmermann E, Deissenrieder F, et al. Noninvasive coronary angiography by 320-row computed tomography with lower radiation exposure and maintained diagnostic accuracy: comparison of results with cardiac catheterization in a head-to-head pilot investigation. *Circulation* 2009; **120**(10): 867-75.

13. Diederichsen AC, Petersen H, Jensen LO, et al. Diagnostic value of cardiac 64-slice computed tomography: importance of coronary calcium. *Scandinavian cardiovascular journal : SCJ* 2009; **43**(5): 337-44.

14. Garcia MJ, Lessick J, Hoffmann MH, Investigators CS. Accuracy of 16-row multidetector computed tomography for the assessment of coronary artery stenosis. *Jama* 2006; **296**(4): 403-11.

15. Ghostine S, Caussin C, Daoud B, et al. Non-invasive detection of coronary artery disease in patients with left bundle branch block using 64-slice computed tomography. *Journal of the American College of Cardiology* 2006; **48**(10): 1929-34.

16. Ghostine S, Caussin C, Habis M, et al. Non-invasive diagnosis of ischaemic heart failure using 64-slice computed tomography. *European heart journal* 2008; **29**(17): 2133-40.

17. Gueret P, Deux JF, Bonello L, et al. Diagnostic performance of computed tomography coronary angiography (from the Prospective National Multicenter Multivendor EVASCAN Study). *The American journal of cardiology* 2013; **111**(4): 471-8.

18. Halon DA, Gaspar T, Adawi S, et al. Uses and limitations of 40 slice multi-detector row spiral computed tomography for diagnosing coronary lesions in unselected patients referred for routine invasive coronary angiography. *Cardiology* 2007; **108**(3): 200-9.

19. Halvorsen BA, Rodevand O, Hagen G, Herud E, Mielczarek W, Molstad P. [Angiography with 64-channel CT upon suspicion of stable coronary disease]. *Tidsskrift for den Norske laegeforening : tidsskrift for praktisk medicin, ny raekke* 2008; **128**(19): 2172-6.

20. Hamdan A, Asbach P, Wellnhofer E, et al. A prospective study for comparison of MR and CT imaging for detection of coronary artery stenosis. *JACC Cardiovascular imaging* 2011; **4**(1): 50-61.

21. Hausleiter J, Meyer T, Hadamitzky M, et al. Non-invasive coronary computed tomographic angiography for patients with suspected coronary artery disease: the Coronary Angiography by Computed Tomography with the Use of a Submillimeter resolution (CACTUS) trial. *European heart journal* 2007; **28**(24): 3034-41.

22. Herzog BA, Husmann L, Burkhard N, et al. Accuracy of low-dose computed tomography coronary angiography using prospective electrocardiogram-triggering: first clinical experience. *European heart journal* 2008; **29**(24): 3037-42.

23. Herzog BA, Wyss CA, Husmann L, et al. First head-to-head comparison of effective radiation dose from low-dose 64-slice CT with prospective ECG-triggering versus invasive coronary angiography. *Heart* 2009; **95**(20): 1656-61.

24. Herzog C, Nguyen SA, Savino G, et al. Does two-segment image reconstruction at 64-section CT coronary angiography improve image quality and diagnostic accuracy? *Radiology* 2007; **244**(1): 121-9.

25. Herzog C, Zwerner PL, Doll JR, et al. Significant coronary artery stenosis: comparison on per-patient and per-vessel or per-segment basis at 64-section CT angiography. *Radiology* 2007; **244**(1): 112-20.

26. Husmann L, Herzog BA, Burger IA, et al. Usefulness of additional coronary calcium scoring in low-dose CT coronary angiography with prospective ECG-triggering impact on total effective radiation dose and diagnostic accuracy. *Academic radiology* 2010; **17**(2): 201-6.

27. Jakamy R, Barthelemy O, Le Feuvre C, et al. Accuracy of multislice computed tomography in the preoperative assessment of coronary disease in patients scheduled for heart valve surgery. *Archives of cardiovascular diseases* 2012; **105**(8-9): 424-31.

28. Jenkins SM, Johnston N, Hawkins NM, et al. Limited clinical utility of CT coronary angiography in a district hospital setting. *QJM : monthly journal of the Association of Physicians* 2011; **104**(1): 49-57.

29. Kajander S, Joutsiniemi E, Saraste M, et al. Cardiac positron emission tomography/computed tomography imaging accurately detects anatomically and functionally significant coronary artery disease. *Circulation* 2010; **122**(6): 603-13.

30. Kefer J, Coche E, Legros G, et al. Head-to-head comparison of three-dimensional navigator-gated magnetic resonance imaging and 16-slice computed tomography to detect coronary artery stenosis in patients. *Journal of the American College of Cardiology* 2005; **46**(1): 92-100.

31. Laissy JP, Messika-Zeitoun D, Serfaty JM, et al. Comprehensive evaluation of preoperative patients with aortic valve stenosis: usefulness of cardiac multidetector computed tomography. *Heart* 2007; **93**(9): 1121-5.

32. Langer C, Peterschroder A, Franzke K, et al. Noninvasive coronary angiography focusing on calcification: multislice computed tomography compared with magnetic resonance imaging. *Journal of computer assisted tomography* 2009; **33**(2): 179-85.

33. Leschka S, Alkadhi H, Plass A, et al. Accuracy of MSCT coronary angiography with 64-slice technology: first experience. *European heart journal* 2005; **26**(15): 1482-7.

34. Leschka S, Scheffel H, Desbiolles L, et al. Combining dual-source computed tomography coronary angiography and calcium scoring: added value for the assessment of coronary artery disease. *Heart* 2008; **94**(9): 1154-61.

35. Leschka S, Scheffel H, Husmann L, et al. Effect of decrease in heart rate variability on the diagnostic accuracy of 64-MDCT coronary angiography. *AJR American journal of roentgenology* 2008; **190**(6): 1583-90.

36. Martuscelli E, Romagnoli A, D'Eliseo A, et al. Accuracy of thin-slice computed tomography in the detection of coronary stenoses. *European heart journal* 2004; **25**(12): 1043-8.

37. Meijboom WB, Meijs MF, Schuijf JD, et al. Diagnostic accuracy of 64-slice computed tomography coronary angiography: a prospective, multicenter, multivendor study. *Journal of the American College of Cardiology* 2008; **52**(25): 2135-44.

38. Meijboom WB, Mollet NR, Van Mieghem CA, et al. Pre-operative computed tomography coronary angiography to detect significant coronary artery disease in patients referred for cardiac valve surgery. *Journal of the American College of Cardiology* 2006; **48**(8): 1658-65.

39. Meijboom WB, Mollet NR, Van Mieghem CA, et al. 64-Slice CT coronary angiography in patients with non-ST elevation acute coronary syndrome. *Heart* 2007; **93**(11): 1386-92.

40. Meijboom WB, Weustink AC, Pugliese F, et al. Comparison of diagnostic accuracy of 64-slice computed tomography coronary angiography in women versus men with angina pectoris. *American Journal of Cardiology* 2007; **100**(10): 1532-7.

41. Mendoza V, Llerena L, Llerena L, Rodríguez L, Olivares E, Linares R. Ischemic heart disease diagnosed by 64 slice computed tomography coronary angiography. *The Internet Journal of Cardiology* 2009; **7**(1).

42. Mollet NR, Cademartiri F, van Mieghem CA, et al. High-resolution spiral computed tomography coronary angiography in patients referred for diagnostic conventional coronary angiography. *Circulation* 2005; **112**(15): 2318-23.

43. Nikolaou K, Knez A, Rist C, et al. Accuracy of 64-MDCT in the diagnosis of ischemic heart disease. *AJR American journal of roentgenology* 2006; **187**(1): 111-7.

44. Nikolaou K, Rist C, Wintersperger BJ, et al. Clinical value of MDCT in the diagnosis of coronary artery disease in patients with a low pretest likelihood of significant disease. *AJR American journal of roentgenology* 2006; **186**(6): 1659-68.

45. Ovrehus KA, Jensen JK, Mickley HF, et al. Comparison of usefulness of exercise testing versus coronary computed tomographic angiography for evaluation of patients suspected of having coronary artery disease. *The American journal of cardiology* 2010; **105**(6): 773-9.

46. Ovrehus KA, Munkholm H, Bottcher M, Botker HE, Norgaard BL. Coronary computed tomographic angiography in patients suspected of coronary artery disease: impact of observer experience on diagnostic performance and interobserver reproducibility. *Journal of cardiovascular computed tomography* 2010; **4**(3): 186-94.

47. Pontone G, Andreini D, Ballerini G, Nobili E, Pepi M. Diagnostic work-up of unselected patients with suspected coronary artery disease: complementary role of multidetector computed tomography, symptoms and electrocardiogram stress test. *Coronary artery disease* 2007; **18**(4): 265-74.

48. Pontone G, Andreini D, Bartorelli AL, et al. Feasibility and accuracy of a comprehensive multidetector computed tomography acquisition for patients referred for balloon-expandable transcatheter aortic valve implantation. *American heart journal* 2011; **161**(6): 1106-13.

49. Pontone G, Andreini D, Bartorelli AL, et al. Diagnostic accuracy of coronary computed tomography angiography: a comparison between prospective and retrospective electrocardiogram triggering. *Journal of the American College of Cardiology* 2009; **54**(4): 346-55.

50. Pontone G, Andreini D, Quaglia C, Ballerini G, Nobili E, Pepi M. Accuracy of multidetector spiral computed tomography in detecting significant coronary stenosis in patient populations with differing pre-test probabilities of disease. *Clinical radiology* 2007; **62**(10): 978-85.

51. Pouleur AC, le Polain de Waroux JB, Kefer J, et al. Usefulness of 40-slice multidetector row computed tomography to detect coronary disease in patients prior to cardiac valve surgery. *European radiology* 2007; **17**(12): 3199-207.

52. Pouleur AC, le Polain de Waroux JB, Kefer J, Pasquet A, Vanoverschelde JL, Gerber BL. Direct comparison of whole-heart navigator-gated magnetic resonance coronary angiography and 40- and 64-slice multidetector row computed tomography to detect the coronary artery stenosis in patients scheduled for conventional coronary angiography. *Circulation Cardiovascular imaging* 2008; **1**(2): 114-21.

53. Pugliese F, Mollet NR, Runza G, et al. Diagnostic accuracy of non-invasive 64-slice CT coronary angiography in patients with stable angina pectoris. *European radiology* 2006; **16**(3): 575-82.

54. Rixe J, Rolf A, Conradi G, et al. Detection of relevant coronary artery disease using dual-source computed tomography in a high probability patient series: comparison with invasive angiography. *Circulation journal : official journal of the Japanese Circulation Society* 2009; **73**(2): 316-22.

55. Rochitte CE, George RT, Chen MY, et al. Computed tomography angiography and perfusion to assess coronary artery stenosis causing perfusion defects by single photon emission computed tomography: the CORE320 study. *European heart journal* 2014; **35**(17): 1120-30.

56. Sato A, Nozato T, Hikita H, et al. Incremental value of combining 64-slice computed tomography angiography with stress nuclear myocardial perfusion imaging to improve noninvasive detection of coronary artery disease. *Journal of nuclear cardiology : official publication of the American Society of Nuclear Cardiology* 2010; **17**(1): 19-26.

57. Scheffel H, Alkadhi H, Plass A, et al. Accuracy of dual-source CT coronary angiography: First experience in a high pre-test probability population without heart rate control. *European radiology* 2006; **16**(12): 2739-47.

58. Shabestari AA, Abdi S, Akhlaghpoor S, et al. Diagnostic performance of 64-channel multislice computed tomography in assessment of significant coronary artery disease in symptomatic subjects. *The American journal of cardiology* 2007; **99**(12): 1656-61.

59. Sun K, Han RJ, Cui LF, et al. Feasibility and Diagnostic Accuracy for Assessment of Coronary Artery Stenosis of Prospectively Electrocardiogram-gated High-pitch Spiral Acquisition Mode Dual-source CT Coronary Angiography in Patients with Relatively Higher Heart Rates: in Comparison with Catheter Coronary Angiography. *Chinese medical sciences journal = Chung-kuo i hsueh k'o hsueh tsa chih* 2013; **27**(4): 213-9.

60. Ugolini P, Pressacco J, Lesperance J, et al. Evaluation of coronary atheroma by 64-slice multidetector computed tomography: Comparison with intravascular ultrasound and angiography. *The Canadian journal of cardiology* 2009; **25**(11): 641-7.

61. Watkins MW, Hesse B, Green CE, et al. Detection of coronary artery stenosis using 40-channel computed tomography with multi-segment reconstruction. *The American journal of cardiology* 2007; **99**(2): 175-81.

62. Xu L, Yang L, Fan Z, Yu W, Lv B, Zhang Z. Diagnostic performance of 320-detector CT coronary angiography in patients with atrial fibrillation: preliminary results. *European radiology* 2011; **21**(5): 936-43.

63. Yang L, Zhang Z, Fan Z, et al. 64-MDCT coronary angiography of patients with atrial fibrillation: influence of heart rate on image quality and efficacy in evaluation of coronary artery disease. *AJR American journal of roentgenology* 2009; **193**(3): 795-801.

64. Unpublished study #1.

65. Unpublished study #2.

*Number of data sets and references may vary. Detailed descriptions of included studies, composition of data sets and number of included patients for analysis can be found in the Supplementary Appendix (Web appendix 2) of the COME-CCT main analysis publication by Haase et al. BMJ 2019;365:l1945.
